# A spatiotemporal proteomic map of human adipogenesis

**DOI:** 10.1038/s42255-024-01025-8

**Published:** 2024-04-02

**Authors:** Felix Klingelhuber, Scott Frendo-Cumbo, Muhmmad Omar-Hmeadi, Lucas Massier, Pamela Kakimoto, Austin J. Taylor, Morgane Couchet, Sara Ribicic, Martin Wabitsch, Ana C. Messias, Arcangela Iuso, Timo D. Müller, Mikael Rydén, Niklas Mejhert, Natalie Krahmer

**Affiliations:** 1 Institute for Diabetes and Obesity, Helmholtz Zentrum München, Neuherberg, Germany; 2 German Center for Diabetes Research (DZD), Neuherberg, Germany; 3 Department of Medicine (H7), Karolinska Institutet, Huddinge, Stockholm, Sweden; 4 Center for Rare Endocrine Diseases, Division of Paediatric Endocrinology and Diabetes, Department of Paediatrics and Adolescent Medicine, Ulm University Medical Centre, Ulm, Germany; 5 Institute of Structural Biology, Molecular Targets and Therapeutics Center, Helmholtz Zentrum München, Neuherberg, Germany; 6 Bavarian NMR Centre, Department of Bioscience, School of Natural Sciences, Technical University of Munich, Garching, Germany; 7 Institute of Neurogenomics, Helmholtz Zentrum München, Neuherberg, Germany; 8 Institute of Human Genetics, Klinikum Rechts der Isar, Technical University of Munich, Munich, Germany; 9 Walther-Straub Institute for Pharmacology and Toxicology, Ludwig-Maximilians-University Munich (LMU), Munich, Germany; 10 Endocrinology unit, Karolinska University Hospital, Huddinge, Stockholm, Sweden

**Keywords:** Mitochondria, Proteomics, Metabolism, Differentiation

## Abstract

White adipocytes function as major energy reservoirs in humans by storing substantial amounts of triglycerides, and their dysfunction is associated with metabolic disorders; however, the mechanisms underlying cellular specialization during adipogenesis remain unknown. Here, we generate a spatiotemporal proteomic atlas of human adipogenesis, which elucidates cellular remodelling as well as the spatial reorganization of metabolic pathways to optimize cells for lipid accumulation and highlights the coordinated regulation of protein localization and abundance during adipocyte formation. We identify compartment-specific regulation of protein levels and localization changes of metabolic enzymes to reprogramme branched-chain amino acids and one-carbon metabolism to provide building blocks and reduction equivalents. Additionally, we identify C19orf12 as a differentiation-induced adipocyte lipid droplet protein that interacts with the translocase of the outer membrane complex of lipid droplet-associated mitochondria and regulates adipocyte lipid storage by determining the capacity of mitochondria to metabolize fatty acids. Overall, our study provides a comprehensive resource for understanding human adipogenesis and for future discoveries in the field.

## Abstract (web summary)

Klingelhuber, Frendo-Cumbo et al. develop a proteomic atlas elucidating the intracellular spatiotemporal changes in protein levels and localizations during human adipogenesis.

## Main

Living organisms have evolved the capacity to store energy in the form of fat in lipid droplets (LDs). The core of these storage organelles contains neutral lipids, such as triglycerides, and an average healthy adult stores 10–25 kg of fat primarily in white adipose tissue (WAT), with each kg equivalent to 9,000 kcal (ref. ^1^).

WAT characterized by few, but large adipocytes (hypertrophy) is associated with insulin resistance and the secretion of pro-inflammatory cytokines, whereas WAT displaying a higher number of small adipocytes (hyperplasia) is linked to a metabolically healthy phenotype^2^. The fact that the balance between hyperplasia and hypertrophy is strongly associated with the risk of developing metabolic complications of obesity underscores the importance of understanding how adipocytes acquire their remarkable capacity for lipid storage and mobilization during differentiation and to identify cellular processes that underlie healthy adipogenesis and lipid dynamics.

Recent technological advances in transcriptomics have substantially improved our understanding of adipocyte heterogeneity and the transcriptional networks underlying adipogenesis^3,4^; however, it has become increasingly clear that post-transcriptional processes are critical for the regulation of protein levels and activity during adipogenesis^5,6^. These dynamic processes, along with the remodelling of organelles, can be understood more thoroughly at the proteomic level. To date, several analyses have provided valuable insights into the protein landscape of adipocytes^7,8^; however, spatiotemporal aspects have not yet been interrogated. Therefore, our current understanding of subcellular reorganization and changes in protein localization during adipocyte differentiation remains incomplete.

To gain insight into how adipocytes reorganize their subcellular structure to achieve their unique capacity for lipid storage, we generated a comprehensive spatiotemporal proteomic map across four different human adipocyte models. Our approach allowed us to determine the proteomic evolution across adipogenesis, which we then compared with primary human white adipocytes and WAT to identify conserved proteomic changes in human adipogenesis. Using a machine-learning-based organelle proteomics approach, we mapped multiple changes in protein localization during adipogenesis. We revealed the coordinated remodelling of metabolic pathways at the level of protein abundance and localization to support de novo lipogenesis, and identified a yet unknown LD protein, C19orf12, previously identified to be associated with mitochondrial membrane protein-associated neurodegeneration (MPAN)^9^ as a regulator of adipocyte function. More specifically, our data show that C19orf12 expression is upregulated during differentiation and localizes to the LD–mitochondrial contact regions, simultaneously interacting with LDs and the mitochondrial import machinery. Depletion of C19orf12 leads to increased LD accumulation due to impaired mitochondrial fatty acid utilization. In human patient cohorts, we found that *C19orf12* expression in WAT was inversely correlated with obesity-associated clinical parameters, which underlies the key role of C19orf12 in human adipocyte lipid storage. Overall, our study offers a comprehensive resource for understanding temporally resolved core proteomic changes in human adipogenesis, as well as the reorganization of organelles and metabolic pathways that drive human adipogenesis.

## Results

### The temporally resolved core proteome of human adipogenesis

**Fig. 1:**
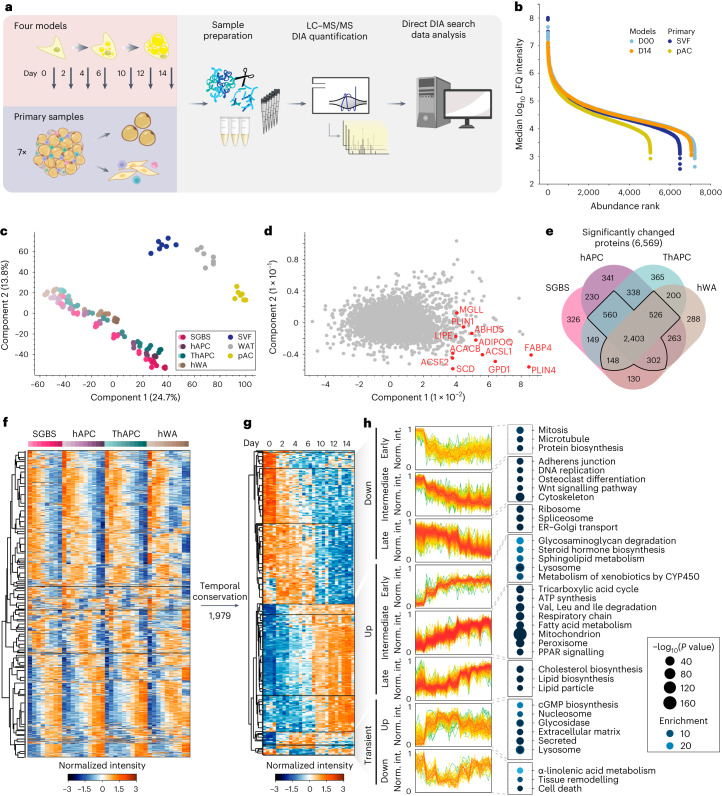
Mapping the temporally resolved core proteome of human adipogenesis. **a**, LC–MS-based proteomics workflow for mapping the core proteome of human adipogenesis. Proteomic signatures of four human adipogenesis models at multiple time points (*n* = 3) were compared with proteomes from human WAT, pACs and SVFs from seven patients. Image was partially created with BioRender.com. **b**, Dynamic range of cell models and primary cell proteomes. **c**, PCA of primary samples and the differentiation stages of the cell lines (depicted as light to dark). Protein filtered for at least two valid values in all cell models and primary samples. **d**, PCA loadings with major driver proteins involved in lipid metabolism shown in red. **e**, Overlap of proteins significantly changed during differentiation in each of the models (individual ANOVA tests for each model, FDR < 10^−2^). **f**, Supervised hierarchical clustering of the *z*-scored temporal profiles of the 3,934 significantly changed proteins in at least three of the four models, as outlined in **e**. **g**, Supervised hierarchical clustering of *z*-scored temporal profiles of all cell models of a subset of **f** with conserved temporal profiles (Pearson correlations of inter-cell model comparison for each protein’s temporal profiles >0). The protein levels of all four models are shown next to each other at the indicated time points. **h**, Profiles of individual clusters and a selection of enriched annotations (one-sided Fisher’s exact test, enrichment score >2, Benjamini–Hochberg FDR < 0.1). Enrichment values and *P* values are depicted as bubble size and colour code, respectively. Source data

To define the core proteome trajectory during human adipogenesis, we performed liquid chromatography–mass spectrometry (LC–MS) proteomics over the time course of differentiation across different human adipocyte models (Fig. 1a). All models are derived from human adipocyte precursor cells (hAPCs) isolated from the stromal vascular fraction (SVF) of WAT and have the capacity to differentiate into adipocytes upon treatment with pro-adipogenic cocktails. Two cell types were non-immortalized (Simpson–Golabi–Behmel syndrome (SGBS)^10^ and hAPC^11,12^), whereas two were immortalized (TERT-hAPC^13^ and hWA^14^). As hAPC and TERT-hAPC are from the same donor, these cells allowed us to control for the potential effects of the immortalization process. Thus, a total of four human model systems were included in this study to map the conserved proteomic landscape of adipogenesis independent of cell type-specific effects. Information regarding the origins, immortalization procedures and differentiation protocols is summarized in Supplementary Tables 1–3. For comparison, we also included primary samples of subcutaneous abdominal mature adipocytes (pACs), SVF (which contains immature adipocyte precursors) and intact WAT from seven donors (Methods). These served as the reference points for in vivo adipogenesis.

**Extended Data Fig. 1:**
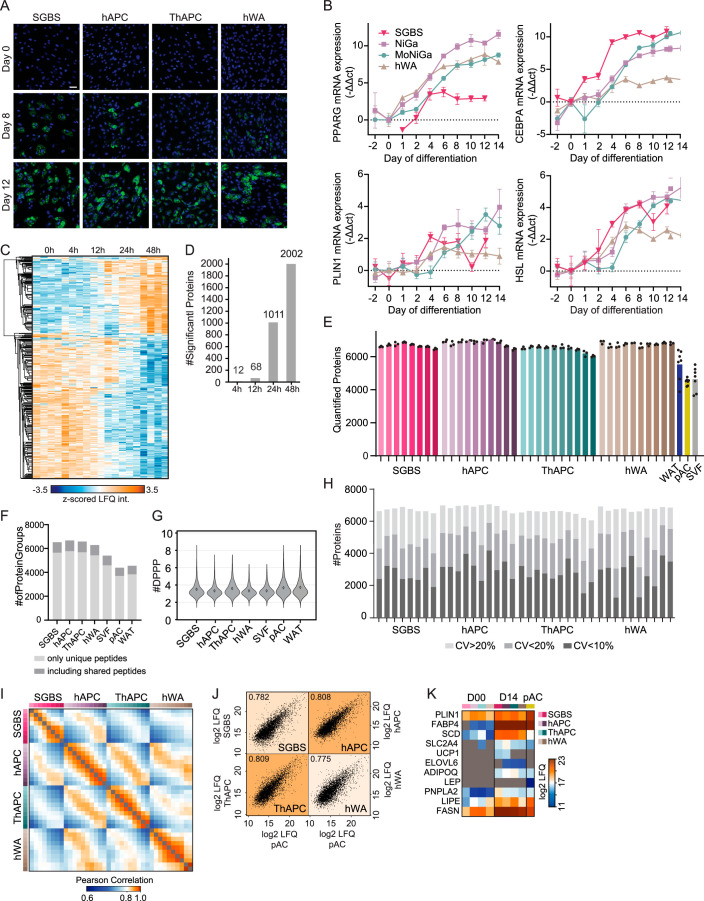
Characterization of human adipogenesis models and comparison with primary cell proteomes. (**a**) Fluorescence microscopy of cell models at early (day 0), middle (day 8), and late stages (days 12–14) of adipogenesis. Experiment repeated twice. LDs stained with BODIPY are shown in green, nuclei stained with DAPI are shown in blue, scale bar = 100µm (n = 3, experiment performed once). (**b**) qPCR of adipogenic marker genes at indicated time points. Values were normalized to the first detected time point. (n = 4, mean ± SD). (**c**) Hierarchical clustering of significantly altered proteins across initial stages of SGBS cell differentiation (n = 4, ANOVA, FDR < 10^(−2)). (**d**) Number of significantly altered proteins in the first 48 h of differentiation (n = 4, two-sided Student′s t-test, FDR < 10^(−2)). (**e**) Number of quantified protein groups during the differentiation of cell models and primary samples (n = 3 for cell models and n = 7 for primary samples, mean ± SD). (**f**) Number of proteins groups identified based only on unique peptides - analysis based on canonical not additional FASTA. (**g**) Violin plot of data points over the peak for each identified peptide in different cell models, average is indicated. (**h**) Number of proteins with the indicated CV values at each time point in each cell model. (**i**) Pearson correlations between samples. (**j**) Pearson correlations of the median protein intensities between each differentiated model and primary adipocytes (minimum two valid per condition, missing values imputed with 0). (**k**) Log2 LFQ intensities of adipogenesis markers in cell models. Source data

First, we assessed the adipogenic capacity of all four models. By measuring lipid accumulation by BODIPY staining followed by fluorescence microscopy and messenger RNA levels of well-established adipogenic marker genes, we found that all models displayed high differentiation efficiencies (Extended Data Fig. 1a,b). We next performed proteome profiling of the cells throughout adipogenesis. Following the induction of differentiation, several proteins changed levels within the first 24 h. The set of early regulated proteins strongly overlays with early adipogenesis markers identified in previous studies (for example upregulation of high-mobility group protein B2 (HMGB2), 11β-hydroxysteroid-dehydrogenase 1 (HSD11B1), FKBP prolyl isomerase 5 (FKBP5) and downregulation of semaphorin 7A (Sema7A))^7,15^. Large-scale remodelling of the proteome could then be observed beginning 24–48 h upon induction of differentiation (Extended Data Fig. 1c,d). Therefore, we analysed the proteomes of the undifferentiated state and during the differentiation, covering time points ranging from 2 to 14 days of differentiation in all cell models. For proteomic analyses, tryptic peptides were analysed in 1-h single shots in the data-independent acquisition (DIA) mode (Methods and Supplementary Table 4). This approach enables higher identification rates over a larger dynamic range and fewer missing values compared with data-dependent acquisition (DDA)^16^. Spectronaut analysis quantified 5,979–7,061 protein groups in the four cell models, 3,638–6,403 in the primary samples (pAC/SVF/WAT) and 8,268 in the complete dataset (Extended Data Fig. 1e) with 86% of proteins being identified based on unique peptides (Extended Data Fig. 1f). The LC–MS signals spanned an abundance range of five orders of magnitude (Fig. 1b). For the majority of quantified peptides (98%), the data points per peak were within the range of 3 to 8 (Extended Data Fig. 1g). At the protein level, 72% of the coefficients of variation (CoVs) were below 20%, and 42% were below 10%, with a median CoV value of 12.3% over all samples (Extended Data Fig. 1h). These CoV values are in close agreement with previously reported numbers for DIA quantification^17^. The high reproducibility of the LC–MS analysis was further confirmed by an average Pearson’s correlation coefficient of 0.97 between replicates (Extended Data Fig. 1i).

**Extended Data Fig. 2:**
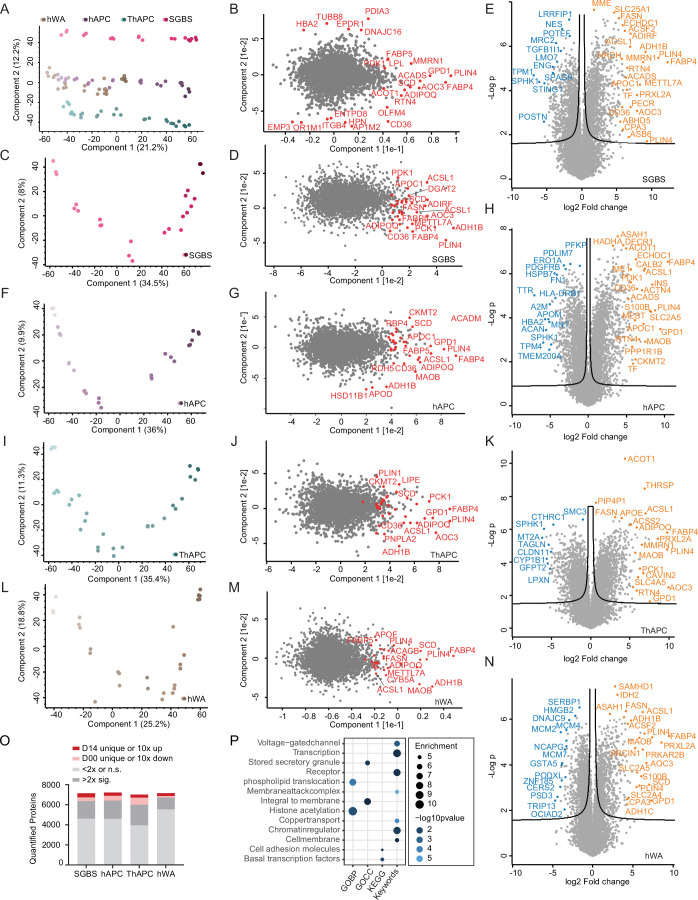
Remodelling of cell model proteomes during adipogenesis. (**a**) PCA of the adipogenic differentiation stages of the cell lines (depicted from light to dark) (filtering for a minimum of 5% valid values across all models, time points, and missing values imputed based on a normal distribution). (**b**) PCA loadings with the major driver proteins involved in lipid metabolism are shown in red. (**c**), (**F**), (**I**), and (**L**) PCAs of adipogenic differentiation stages of each cell model. (**d**), (**G**), (**J**), and (M) PCA loadings with the major driver proteins involved in lipid metabolism are shown in red for each cell model. (**e**), (**H**), (**K**), and (**N**) volcano plots for each cell model comparing the proteomes of preadipocytes and adipocytes from day 14 (n = 3, FDR < 10^(−2) and S0 = 0.1). The most strongly upregulated or downregulated proteins are indicated in orange and blue, respectively. (**O**) Number of proteins quantified during differentiation in each cell model (min. two detections in at least one time point), and the number of significantly regulated proteins or proteins exclusively quantified in either mature adipocytes or preadipocytes (two-sided Student’s t-test, FDR < 10^(−2)). (**P**) Enrichment analysis for GO-terms, keywords, and KEGG pathways among proteins of the stable proteome during adipogenesis (one-sided Fisher’s exact test, enrichment score >2, Benjamini-Hochberg FDR < 0,1). Source data

When comparing the samples, the Pearson correlation coefficients were high within the same time points between the systems (Extended Data Fig. 1j), indicating that there are shared proteomic features of adipogenesis that can be recapitulated in multiple in vitro models. This enabled us to identify the universal events of human adipogenesis, which are not dependent on donors and are not affected by immortalization. Principal-component analysis (PCA) confirmed that the proteomic data recapitulated the cellular transition along the differentiation trajectory in vitro (the four cell models) and in vivo (SVF versus pACs) in components 1 and 2, where the adipogenic process of the cell lines projected towards mature adipocytes (Fig. 1c). An increase in LD and lipid biosynthesis protein levels was a key factor driving the trajectory along component 1 (Fig. 1d). Moreover, our PCA confirmed that hWA cells reached a less-mature state than the other models, as indicated by microscopy and mRNA/protein levels of adipocyte markers (Extended Data Fig. 1a,b,k). Additional PCAs conducted exclusively on the cell models, both collectively and individually, confirmed that the most prominent separation in component 1 was associated with the differentiation process and primarily driven by elevated levels of numerous proteins linked to lipid synthesis (Extended Data Fig. 2a–n).

Our proteomic analysis based on normalization to equal protein input per condition and sample, found that a considerable proportion (38%) of the proteome underwent remodelling during the differentiation process (analysis of variance (ANOVA), false discovery rate (FDR) 0.01) in all four models. About 6–14% of the quantified proteins showed a more than tenfold change compared with the undifferentiated state or were exclusive to either the undifferentiated or mature state (Extended Data Fig. 2o). Out of the 3,939 proteins with significantly altered level in at least three of the four models (Fig. 1e,f), approximately half of them (*n* = 1,979) displayed a conserved temporal trajectory during adipogenesis, which we defined as a positive correlation between the temporal profiles in all pairwise comparisons between the cell models. Next, we performed a supervised hierarchical clustering analysis on the conserved temporal profiles and identified distinct clusters representing early, intermediate and late responses during adipogenesis (Fig. 1g,h). The early phase was characterized by the downregulation of proteins involved in cell cycle progression and protein biosynthesis, as well as upregulation of glycosaminoglycan degradation and lysosomal pathways. The intermediate phase was defined by a significant increase in enzymes involved in fatty acid metabolism and mitochondria-related functions, such as the tricarboxylic acid (TCA) cycle, respiratory chain and adenosine triphosphate (ATP) synthesis. Simultaneously, the levels of proteins involved in DNA replication, cytoskeletal and cell adhesion proteins, and WNT signalling (a pathway that inhibits adipogenesis^18^) decreased. In the late phase of adipogenesis, we observed downregulation of spliceosomes and mRNA-processing proteins and upregulation of cholesterol biosynthesis and several LD proteins. Considering the large-scale proteome remodelling, we explored whether there are shared functions among proteins that exhibited no changes in abundance during adipogenesis. Out of the 1,500 proteins that remained unaltered across all four models, we observed a significant enrichment of fundamental cellular machineries, including transcription factors, chromatin modifiers and plasma membrane transporters (Extended Data Fig. 2p).

**Extended Data Fig. 3:**
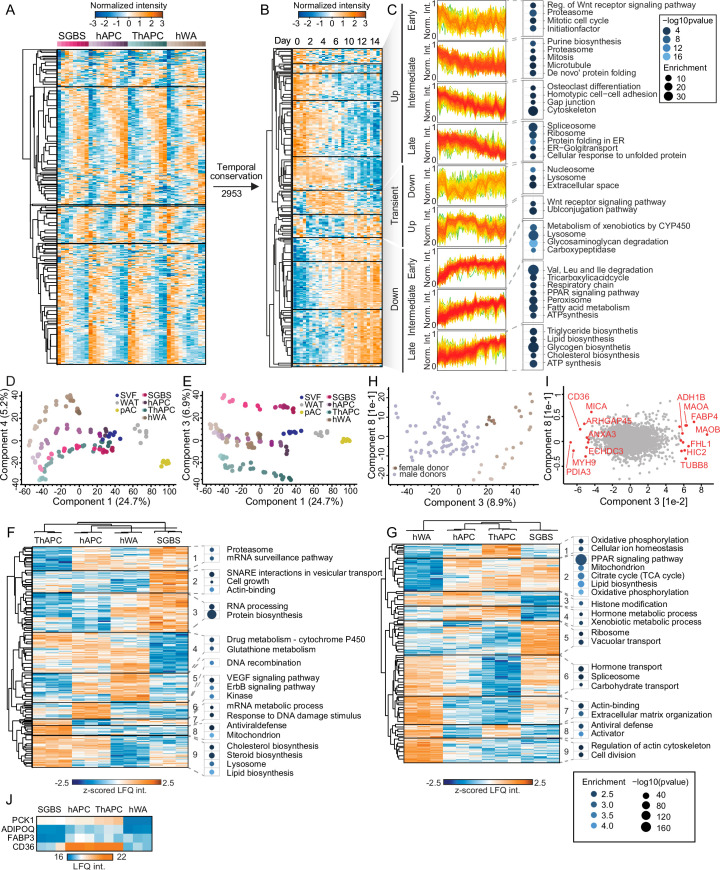
Changes in protein copy number and differences between the cell models. (**a**) Supervised hierarchical clustering of the z-scored temporal profiles of ranked copy numbers of significantly changed proteins in at least three of the four models (n = 3, ANOVA conducted on copy numbers, FDR < 10^(−2)). (**b**) Supervised hierarchical clustering of z-scored temporal profiles of all cell models of a subset of (A) with conserved temporal profiles (Pearson correlation of z-scored temporal profiles in all inter-cell model comparisons >0). The copy numbers of all the four models are shown next to each other at each indicated time point. (**c**) Representative GO-annotations, KEGG pathways, and Keywords enriched in the indicated clusters are shown. Enrichment values and p values are depicted as bubble size and colour code, respectively (one-sided Fisher’s exact test, enrichment score >2, Benjamini-Hochberg FDR < 0,1). (**d**) and (**e**) PCAs of the adipogenic differentiation stages of the cell lines (depicted from light to dark) showing components 3 and 4. (**f**) and (**g**) Unsupervised hierarchical clustering of z-scored protein levels of significantly different proteins between preadipocytes and adipocytes on days 0 and 14 across the four cell models, respectively (n = 3, ANOVA, FDR < 0.01). Representative GO-annotations, KEGG pathways, and Keywords enriched in the indicated clusters are shown (one-sided Fisher’s exact test, enrichment score >2, Benjamini-Hochberg FDR < 0,1). P values and enrichment scores are indicated by bubble colour and size (**h**) PCA showing Components 3 and 7, separating cell models of male and female donors. (**i**) PCA loading showing the major driver proteins of Component 3 in red. (**j**) Heatmap of LFQ intensities of PPARγ targets on day 14 (n = 3). Source data

By using the proteomic ruler^19^, which provides an absolute scale for the LC–MS readout by measuring the protein copy numbers per cell through the intensity of histones (assumed to be proportional to the amount of DNA and thereby the number of cells in the sample), we further mined our data (Extended Data Fig. 3a–c). Supervised hierarchical clustering of temporal profiles of the ranked copy numbers and a subsequent functional annotation enrichment analysis yielded a high overlap compared with our analyses normalized to the same protein amounts, suggesting a comprehensive remodelling of cellular processes during adipogenesis, with distinct temporal regulation of specific functional pathways independent from the normalization method.

### Similarities and differences between cell models

The results of the PCA of the cell models and clinical samples demonstrated that components 3 and 4 effectively separated the individual cell models (Extended Data Fig. 3d,e). To gain a more comprehensive understanding of the distinctions between the cell models, we conducted statistical analyses in both undifferentiated and fully differentiated states. Unsupervised clustering analysis of the significantly different proteins (ANOVA, FDR < 0.01) revealed unique expression patterns among the cell models in preadipocytes and at the end of adipogenesis. In the undifferentiated state, SGBS cells exhibited the most distinctive profile, characterized by elevated ribosomal and reduced proteasomal protein levels (Extended Data Fig. 3f, cluster 1). Among the differentiated models, hWA cells displayed the most distinct features (Extended Data Fig. 3g), primarily associated with their less-mature state. This was supported by the upregulation of a cluster related to cell cycle and cytoskeletal proteins (Extended Data Fig. 3g, cluster 9), and downregulation of a separate cluster containing mitochondrial and LD proteins (Extended Data Fig. 3g, cluster 2). Furthermore, we observed features in hWA cells derived from a 48-year-old female donor that could be attributed to sex- or age-specificity, whereas the other cell models were derived from young male donors (SGBS from a newborn and hAPCs/TERT-hAPCs from a 16-year-old). Specifically, cluster 9 (Extended Data Fig. 3g), upregulated in hWAs, exhibited heightened levels of monoamine oxidases A and B (MAOA and MAOB), which regulate norepinephrine degradation and consequently control lipolysis, along with alcohol dehydrogenase 1B (ADH1B). These proteins emerged as the primary drivers of component 3 in a PCA, effectively distinguishing hWAs from other cell models (Extended Data Fig. 3h–i). Previous studies have underscored the significant upregulation of these three enzymes in female adipocytes during aging^20^. Another obvious difference between the cell models was that TERT-hAPCs and hWAs, which underwent an immortalization procedure, displayed upregulation of proteins associated with viral defence mechanisms (Extended Data Fig. 3f, cluster 8 and Extended Data Fig. 3g, cluster 8). We did not observe clear effect attributed to the distinct treatment protocols, except for the higher levels of peroxisome proliferator-activated receptor γ (PPARγ) targets in cell models that were treated with the PPARγ agonist rosiglitazone for a longer duration (Extended Data Fig. 3j). In summary, the temporally resolved proteomic characterization revealed that essential features are consistently preserved across various models, yet distinct features are evident and linked to age, sex and the process of immortalization.

### Deviations in temporal dynamics between transcriptome and proteome

**Extended Data Fig. 4:**
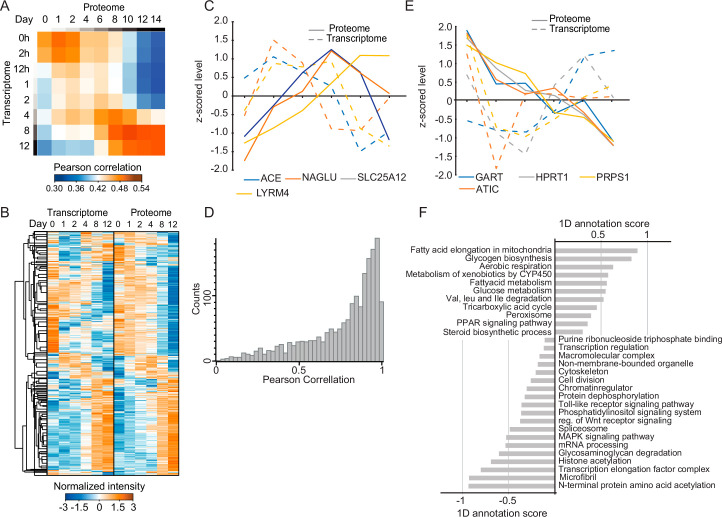
Temporal changes in the proteome and transcriptome during adipogenesis. (**a**) Pearson correlation coefficients between protein and mRNA levels at each indicated time point of differentiation in hAPCs. The datasets were filtered for the temporally conserved core proteome from (1H). (**b**) Supervised hierarchical clustering of z-scored profiles at protein and mRNA levels. (**c**) Z-scored protein and mRNA profiles of indicated proteins. (**d**) Histogram of Pearson correlation values between the temporal protein and mRNA profiles of proteins, including all time points present in both datasets. (**e**) Z-scored protein and mRNA profiles of indicated proteins. (**f**) 1D annotation scores of representative GO-terms, keywords, and KEGG pathways enriched among pathways with high and low temporal mRNA and protein correlations (two-sided 1D annotation enrichment, Benjamini-Hochberg FDR < 0.1). Source data

To test whether the regulation of the adipogenic core proteome is determined at the transcriptional level, we integrated the proteome of hAPCs with a previously generated transcriptomic time course of the same cell model^21^. The correlation between the two datasets was between 0.39–0.48 at the individual time points and increased at the later stages (Extended Data Fig. 4a). A comprehensive comparison revealed that as anticipated, modifications to the proteome typically lagged those of the transcriptome, albeit the extent of this delay varied among individual proteins (Extended Data Fig. 4b,c). 16% of the temporal protein profiles displayed a negative correlation with corresponding mRNA profiles (Extended Data Fig. 4d), as shown here for the mRNA and proteins profiles of proteins involved in purine synthesis (Extended Data Fig. 4e). Overall, metabolic pathways showed a higher correlation between protein and mRNA abundance than proteins involved in signalling pathways, chromatin regulation and transcriptional regulation. Additionally, proteins involved in protein complexes and non-membrane-bound organelles showed low correlation, indicating enhanced regulation at the protein level (Extended Data Fig. 4f). Thus, our results indicate that other aspects in addition to gene transcription, such as protein degradation, contribute to changes in protein abundance during adipogenesis. mRNA can only be used as a proxy for protein abundance to some extent and does not comprehensively reveal temporal dynamics at the protein level.

### A spatial proteome map of adipogenesis

To add a spatial dimension to our proteome map of adipogenesis, we utilized protein correlation profiling (PCP), a technique that allows for the analysis of organellar protein localization based on relative abundance profiles^22^. Using this approach, the determination of protein localization does not rely on obtaining pure organelle fractions, but rather on the characteristic behaviour of proteins associated with specific compartments during the fractionation process. In brief, for PCP, cells are mechanically lysed and the organelles are separated by density-gradient centrifugation. Next, proteins are quantified across gradient fractions by LC–MS to generate abundance profiles. These, in turn, are highly characteristic of the residual cellular compartments, reflect potential multiple organellar localizations and can subsequently be used to predict protein localization by machine learning^23^.

**Fig. 2:**
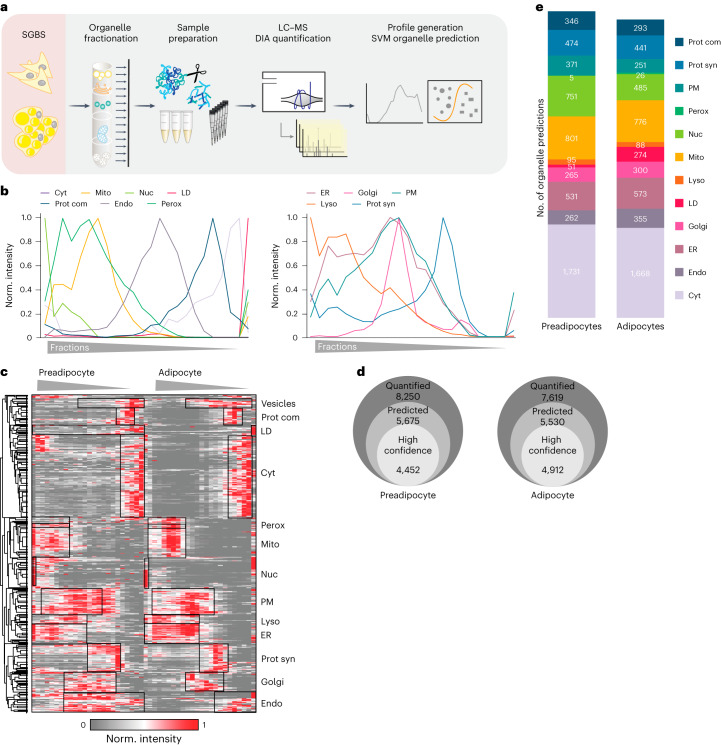
Generation of a cellular map of human adipogenesis. **a**, Generation of a human adipocyte organellar map by PCP. Either undifferentiated or fully differentiated SGBS cells were lysed and the organelles were separated. Organelle fractions were analysed by DIA-LC–MS. Protein profiles were generated and SVM-machine learning was used to predict protein localizations. **b**, Median profiles from biological triplicates for indicated organelles in mature adipocytes based on all proteins assigned to an organelle with a single localization. **c**, Supervised hierarchical clustering of protein profiles (median of triplicates) from preadipocytes and adipocytes filtered for inter-replicate Pearson correlations >0. GO terms for organelles enriched in the marked clusters are highlighted (one-sided Fisher’s exact test, enrichment score >2, Benjamini–Hochberg FDR < 0.1). Cyt, cytosol; Perox, peroxisome; Mito, mitochondrion; Nuc, nucleus; PM, plasma membrane; Lyso, lysosome; Prot syn, protein synthesis; Endo, endosome. **d**, Numbers of quantified and predicted proteins in preadipocytes and in adipocytes. **e**, Numbers of proteins assigned to organelles as first association by SVM-based learning on concatenated protein profiles (*n* = 3 for preadipocytes, *n* = 4 for adipocytes). Source data

**Extended Data Fig. 5:**
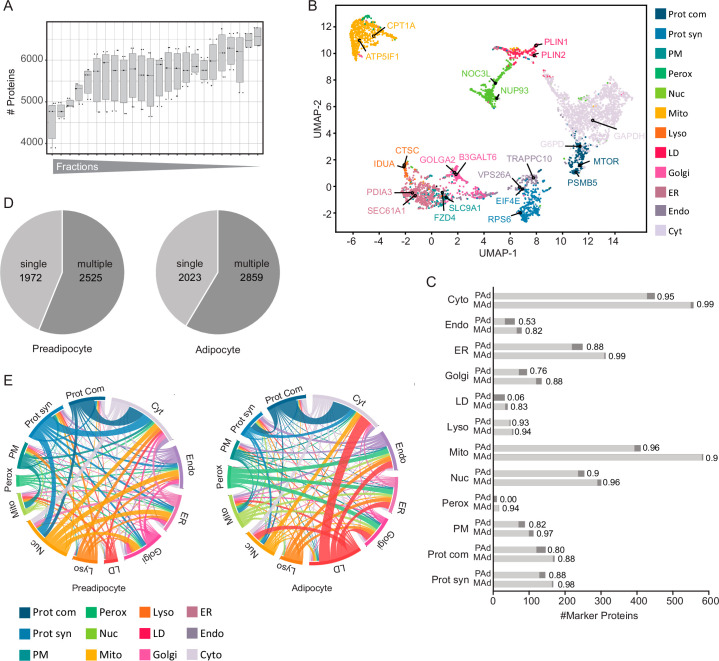
A cellular map of human preadipocytes and adipocytes. (**a**) Boxplot showing the number of quantified proteins per organelle fraction in adipocytes and preadipocytes (n = 7, error bars spread from min to max, box extends from the 25th to 75th percentile, line represents the median). (**b**) UMAP visualization of adipocyte PCP dataset. Proteins are coloured according to their organelles. Names of canonical markers are indicated. (**c**) The number of organelle markers per compartment and prediction accuracy of these markers by SVMs for preadipocytes (pAd) and mature adipocytes (mAd). (**d**) Number of proteins with single- and dual-protein predictions in the preadipocytes and adipocytes. (**e**) Circular plots of the first and second organelle predictions for preadipocyte and adipocyte organelle maps. Each Circos plot depicts the location of the first organelle in the outer ring and the second organelle in the inner ring. The second organelle is indicated in colour. Each connection line displays a combination of dual localizations, as revealed by the organelle map. The colour of the connection line corresponds to the first organelle assignment. Source data

To identify proteins that display different locations during adipogenesis, we applied PCP to differentiated adipocytes and preadipocytes using the SGBS model (Fig. 2a and Supplementary Table 5). By conducting 1-h LC–MS DIA single shot analyses, we achieved quantification of 3,500–5,600 proteins per fraction (Extended Data Fig. 5a), resulting in cellular maps with increased proteomic coverage and identification rates, less LC–MS runtime and higher reproducibility compared with traditional DDA-based approaches^16^. While there was some overlap, we were able to differentiate between organelles based on their respective profiles. This was possible because each compartment, while sharing certain regions of density-gradient overlap, also exhibited distinctive regions where they deviated from one another (Fig. 2b). One exception in preadipocytes was peroxisomes, which co-floated with the endoplasmic reticulum (ER) in preadipocytes, rendering accurate assignment to this organelle impossible. Therefore, we excluded peroxisomes for the subsequent downstream analysis on protein localization changes. Supervised hierarchical clustering and a Uniform Manifold Approximation and Projection (UMAP) visualization of the median profiles from the biological replicates indicated distinct clusters for most cellular compartments with canonical marker proteins grouping with other members based on their similarity (Fig. 2c and Extended Data Fig. 5b).

By employing support vector machine (SVM)-based supervised learning, we were able to predict primary and potential secondary protein localization using the generated abundance profiles. Organellar cluster boundaries were determined using proteins with experimentally validated localizations. These markers were extracted from a proteomic organelle map curated from HeLa cells^24^ and microscopy-derived localizations across various cell lines based on the Protein Atlas^25^. To further enhance the marker set, especially for LDs that are sparsely covered in these datasets, we supplemented our analyses with proteins localizing to LDs in multiple datasets of the Lipid Droplet Knowledge Portal (LDKP)^26^. Based on these data, we achieved a mean prediction accuracy of 96% for adipocytes and 88% for preadipocytes. The lower accuracy observed in preadipocytes stems from the absence of LDs in this cell type and the consequent assignment of LD marker proteins to other compartments (Extended Data Fig. 5c). When examining prediction accuracy across different organelles, we observed that mitochondrial and cytoplasmic markers achieved the highest level of accuracy, whereas markers for endosomes and LDs exhibited relatively lower accuracy levels of 88% in adipocytes (Extended Data Fig. 5c). This most probably reflects the dynamic nature and non-exclusive localization of these compartment-resident proteins.

In brief, our mapping of adipocytes and preadipocytes reveals the localization of a total of 5,530 and 5,675 proteins, respectively. Within these, 4,452 proteins in adipocytes and 4,912 proteins in preadipocytes were assigned to specific organelle clusters by SVMs with high confidence (Fig. 2d,e). Over half of the proteins were found to be associated with at least two organelles, (Extended Data Fig. 5d,e), aligning with observations from previous studies^27^. Consequently, we offer a comprehensive reference on protein localization and alterations during adipogenesis.

### Protein localization changes in adipogenesis

**Fig. 3:**
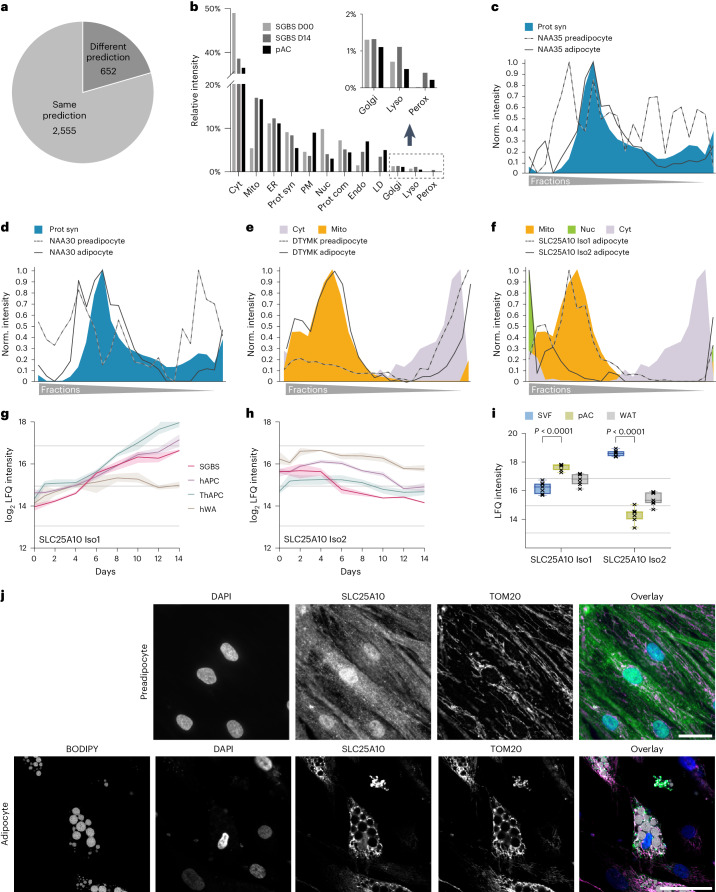
Changes in protein localization during adipogenesis. **a**, The number of proteins assigned with high confidence to the same or different compartments in SGBS preadipocytes and adipocytes. **b**, Percentage of organelles in the total proteome of preadipocytes (SGBS day 0), mature adipocytes (SGBS day 14) and pACs based on integration of the first organelle assignments and summed protein-LFQ intensities in the total proteome analysis. **c**,**d**, Profiles of subunits of the NATC complex in preadipocytes and adipocytes overlaid with the respective organelle marker profiles. **e**, DTYMK profile of preadipocytes and adipocytes overlaid with the respective organelle marker profiles. **f**, Profiles of the two detected isoforms of SLC25A10 in adipocytes overlaid with the mitochondrial, cytosolic and nuclear marker profiles. **g**,**h**, Temporal profile of LFQ intensities of SLC25A10 isoforms from four cell models during adipogenesis (lines represent mean and light areas the whole range). **i**, SLC25A10 isoform levels in SVFs, pACs and WAT (two-sided, paired Student’s *t*-tests, FDR < 0.05, error bars spread from min to max, box extends from 25th to 75th percentile, line represents median, *n* = 7). **j**, Representative immunofluorescence of SLC25A10 in hAPC preadipocytes and adipocytes. DAPI is shown in blue, BODIPY in grey, SLC25A10 in green and TOM20 in magenta. Scale bar, 50 µm. Representative images from three conducted experiments for both hAPC preadipocytes and adipocytes. Source data

**Extended Data Fig. 6:**
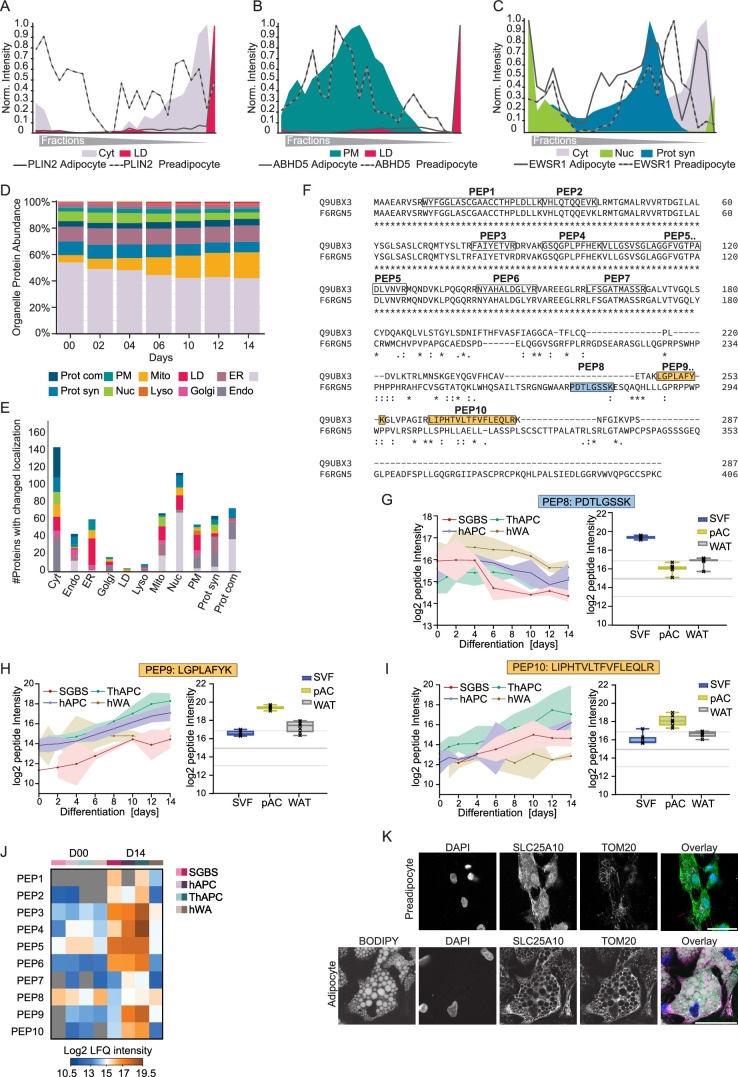
Organelle remodelling during adipogenesis. (**a**)–(**c**) Examples of protein profiles previously reported to change localization during adipogenesis. The protein profiles of the preadipocytes and adipocytes were overlaid with the average organelle marker proteins of the indicated compartments. (**d**) Percentage of organelle intensities in the total proteomes during the time course of differentiation. Calculations were based on the first localization of the assigned proteins in either preadipocytes or adipocyte PCP under the respective conditions. For intermediate time points, quantification was based on proteins with the same localization under both conditions. (**e**) Frequency of changes in localization between preadipocytes and adipocytes in each organelle for proteins assigned with high confidence. Localization in the preadipocytes is displayed on the x-axis, and the number of localization changes is displayed on the y-axis. Target organelles are indicated by their respective colours. (**f**) Amino acid sequence alignment of the two SLC25A10 isoforms. Common and isoform-specific precursors are marked. (**g**), (**h**), and (**i**) Temporal profiles of the intensities of the indicated precursors in the cell models (n = 3, mean ± 95% confidence interval) and their LFQ intensities in the primary cells (n = 7, error bars from boxplot spread from min to max, box extends from the 25th to 75th percentile, line represents median). (**j**) Heatmap of z-scored profiles of the indicated precursors in the preadipocytes, adipocytes, and primary cells. (**k**) Immunofluorescence of SLC25A10 in SGBS preadipocytes and adipocytes. DAPI is shown in blue, BODIPY in grey, SLC25A10 in green, and TOM20 in magenta. Scale bar = 50µm. Representative images of two experiments in preadipocyte and three in adipocytes. Source data

Based on our SVM analysis, 1,323 proteins displayed divergent organelle assignments comparing undifferentiated progenitor cells and differentiated adipocytes, among those 654 with high confidence in both datasets (Fig. 3a). We mined this list for proteins known to undergo changes during adipogenesis and found perilipin2 (PLIN2; a member of the perilipin family) and abhydrolase domain containing 5 (ABHD5; the cofactor of adipose triglyceride lipase) to exhibit previously reported localization shift towards LDs^28,29^ (Extended Data Fig. 6a,b). Furthermore, our data confirmed the known nuclear translocation of the RNA-binding protein Ewing sarcoma breakpoint region 1 (EWSR1)^30^ (Extended Data Fig. 6c).

Next, we leveraged these spatial proteomics data and the time-resolved core proteome of adipogenesis to characterize organelle remodelling during adipogenesis. By integrating information from both datasets, we were able to predict the proportion of each organelle in the total proteome. Our findings showed that, during adipogenesis, there was an increase in the percentage of mitochondrial, ER, endosomal and LD proteins, whereas the proportion of cytosolic and nuclear proteins decreased. These changes in organelle composition reflected an overall increase in the total protein mass of all compartments involved in lipid metabolism and secretory functions, which ultimately led to a state that closely resembled the proportional organelle distribution in pACs (Fig. 3b and Extended Data Fig. 6d).

Through our analysis, we identified protein exchange between different cellular compartments (Extended Data Fig. 6e). Compared to the size of their proteomes, compartments of the vesicular trafficking pathway such as endosomes and the plasma membrane were particularly affected, as well as proteins complexes and the translational machinery. For instance, we observed a reorganization of the translational machinery during adipogenesis. In mature adipocytes, the N-terminal acetyltransferase (NAT)C complex was associated with the translational machinery, whereas in preadipocytes, the subunits of the same complex exhibited a diffuse distribution across all fractions (Fig. 3c,d). Indeed, NATs can bind to ribosomes where they perform N-terminal acetylation in a co-translational manner to regulate protein degradation rates and interactions^31^. Notably, among NAT complexes, NATC is particularly important to modify mitochondrial proteins, which are strongly induced in adipogenesis^32^. As another example for a protein localization change, we mapped the translocation of numerous mitochondrial proteins, including deoxythymidylate kinase (DTYMK), which is involved in pyrimidine biosynthesis. During adipogenesis, DTYMK displayed increased mitochondrial targeting, with a concomitant decrease in the cytosol (Fig. 3e).

Additionally, we observed an alternative mechanism contributing to changes in protein localization during adipogenesis, which involved the regulation of expression of isoforms with distinct localizations. This phenomenon was observed for SLC25A10, the mitochondrial dicarboxylate carrier responsible for succinate transport and predominantly expressed in WAT^33^. During adipogenesis, isoform 2 with a nuclear profile was downregulated, whereas isoform 1 with mitochondrial localization substantially increased (Fig. 3f,h). This isoform switch was present in all four cell models and reflected in primary adipocytes compared with the SVF (Fig. 3i). Among the detected peptides, two were unique to isoform 1 and one was unique to isoform 2 (Extended Data Fig. 6f). The profiles of the unique peptides further confirmed the decrease in isoform 2 and the increase in isoform 1 during differentiation (Extended Data Fig. 6g–i). A strong and uniform increase in the shared peptides despite the decrease in isoform 2-specific peptide, indicated that most of the newly formed protein belongs to the mitochondrial isoform 1 (Extended Data Fig. 6j). We further confirmed the isoform switch-driven localization change through immunofluorescence staining of SLC25A10 using an antibody recognizing both isoforms in hAPCs and SGBS cells (Fig. 3j and Extended Data Fig. 6k). The staining showed SLC25A10 localization in the nucleus and cytosol of preadipocytes and in the mitochondria of adipocytes. In summary, our findings highlight that approximately 20% of the mapped proteome change their localization during adipogenesis, indicating a critical role for the regulation of protein localization in cellular differentiation processes.

### Protein localization and abundance changes drive cell reprogramming

**Fig. 4:**
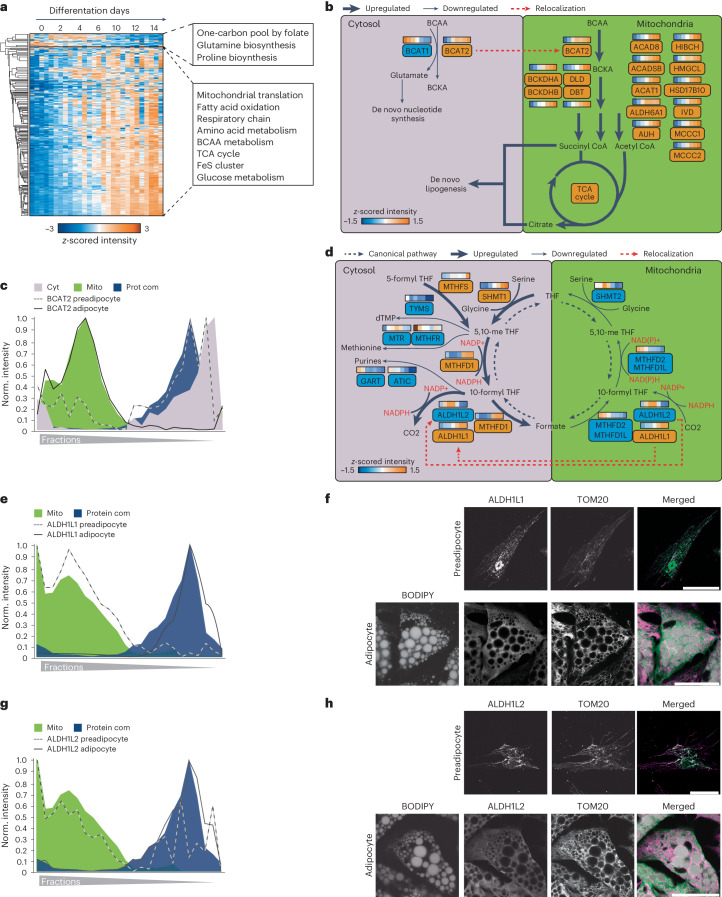
Integration of spatial proteomics with protein levels to characterize organelle metabolic reprogramming. **a**, Hierarchical clustering of significantly changed *z*-score protein profiles in the total proteome over the differentiation time course, for all proteins predicted to be mitochondrial. The values from the four cell models were sorted next to each other at the same time point. GO terms enriched in the clusters compared with the total mitochondrial proteome are highlighted. **b**, Scheme of BCAA metabolism and its changes during adipogenesis. Upregulated and downregulated proteins are marked in orange and blue, respectively. The colour codes in the boxes display the median protein levels during adipogenesis across all cell models. Thin arrows indicate downregulated reactions and thick pathways indicate upregulated reactions during adipogenesis. The change in the protein localization of BCAT2 during adipogenesis is indicated by a red arrow. Figure were created with BioRender.com. **c**, Protein profile of BCAT2 and indicated organelle marker profiles. **d**, Scheme of one-carbon metabolism remodelling during adipogenesis. Upregulated and downregulated proteins are marked in orange and blue, respectively. The colour code in the boxes displays the median protein levels during adipogenesis across all cell models. The direction of the canonical pathway in proliferating preadipocytes is indicated by the grey arrow. Protein translocations of ALDH1L1 and ALDH1L2 are indicated by red arrows. The predicted flux change and reversal based on protein levels and localization are indicated by the arrows. The downregulated mitochondrial part of the cycle is indicated by a thin arrow and the potentially reversed and upregulated cytosolic part of the cycle is indicated by a thick arrow. The figure was created with BioRender.com. **e**,**g**, Protein profiles of ALDH1L1 and ALDH1L2 and indicated organelle marker profiles. **f**,**h**, Representative immunofluorescence staining for ALDH1L1 and ALDH1L2 in SGBS preadipocytes and adipocytes, respectively. In the overlay, BODIPY is shown in grey, ALDH1L1 and ALDH1A2 are in green and TOM20 is in magenta. Scale bar, 50 µm. Representative images from three experiments are shown. Source data

**Extended Data Fig. 7:**
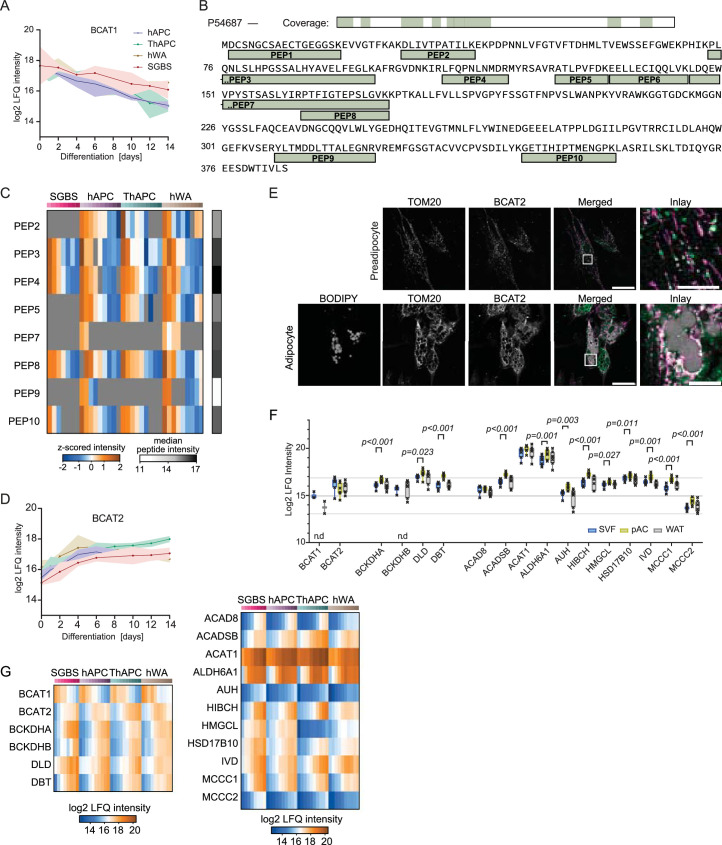
BCAA catabolism reprogramming during adipogenesis. (**a**) Log2 LFQ intensities of BCAT1 in four adipogenesis models during differentiation (n = 3, lines represent mean and light areas the entire range). (**b**) Amino acid sequence of BCAT1. Quantified peptides are shown. (**c**) Temporal profiles of BCAT1 peptides (n = 3, mean ± 95% confidence interval). (**d**) Log2 LFQ intensities of BCAT2 in four adipogenesis models during differentiation (n = 3, lines represent mean and light areas the entire range). (**e**) Immunofluorescence of BCAT2 in hAPC preadipocytes and adipocytes. In the overlay, BODIPY is shown in grey, BCAT2 in green, and TOM20 in magenta. Scale bar = 50µm in overlay and 10µm in inlay. The experiment was repeated four times. (**f**) Log2 LFQ intensities of BCAA metabolism enzymes in primary samples (n = 7, two-sided paired Student’s t-tests, FDR < 0.05, error bars spread from min to max, box extends from the 25th to 75th percentile, line represents the median). (**g**) Heatmaps of log2 LFQ intensities of BCAA metabolic enzymes in all four models during differentiation (median, n = 3). Source data

To gain a better understanding of how organelles respond during adipogenesis, we conducted cluster analysis of temporal protein profiles assigned to specific organelles as exemplified here for mitochondria. Our analysis indicated that the notable increase in the total amount of mitochondrial protein during adipogenesis (Fig. 3b) was accompanied by the upregulation of various mitochondrial pathways, including the TCA cycle, respiratory chain complexes and branched-chain amino acid (BCAA) catabolism (Fig. 4a), consistent with previous findings that degradation of the amino acids valine, leucine and isoleucine provides an essential pool of acetyl-CoA for de novo lipogenesis in adipocytes^34^. Our spatiotemporal data integration revealed compartment-specific regulation of both the levels and localization of BCAA catabolism enzymes (Fig. 4b). Specifically, we found that during the proliferative phase of adipocyte precursor cells, BCAT1 and BCAT2, the first enzymes in the BCAA degradation pathway, are in the cytosol (Fig. 4b–c). This localization enables the degradation of BCAA to produce glutamine, a key component required for de novo nucleotide biosynthesis, which is critical for cell division; however, during differentiation, branched-chain amino acid transaminase 1 (BCAT1) was downregulated (Extended Data Fig. 7a). The downregulation was evident at protein and individual peptide levels (Extended Data Fig. 7b,c). At the same time, branched-chain amino acid transaminase 2 (BCAT2) was upregulated (Extended Data Fig. 7d) and translocated to the mitochondria as indicated by our PCP (Fig. 4c) and confirmed by immunofluorescence in hAPCs (Extended Data Fig. 7e). BCAT1 downregulation and BCAT2 upregulation during adipogenesis were also evident in primary cells (Extended Data Fig. 7f). As all mitochondrial BCAA metabolism enzymes increase their levels during differentiation (Extended Data Fig. 7f,g), we hypothesize that the upregulation of these enzymes, coupled with BCAT2 translocation to the mitochondria, shifts the pathway from cytosol to mitochondrial BCAA degradation, leading to the production of acetyl-CoA via the TCA cycle, which largely fuels de novo lipogenesis in adipogenesis^34^.

**Extended Data Fig. 8:**
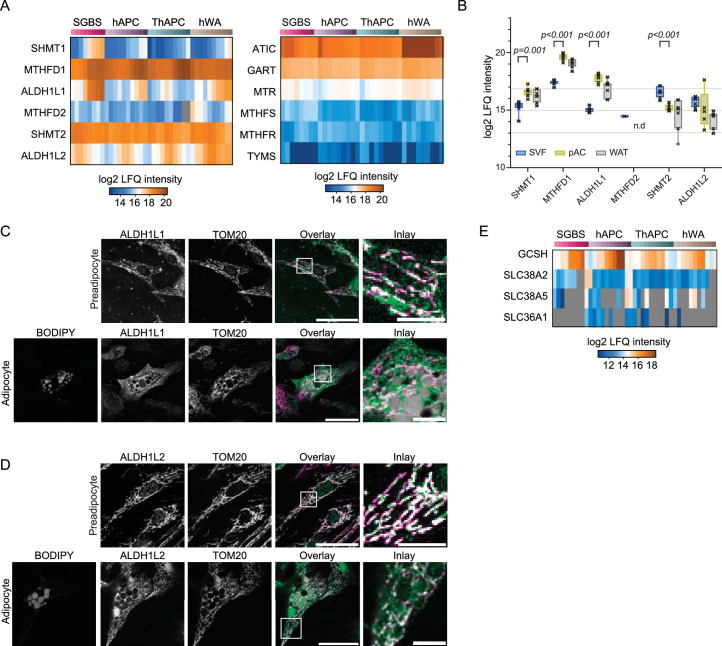
Reprogramming of the one-carbon cycle during adipogenesis. (**a**) Heatmap of log2 LFQ intensities of one-carbon cycle enzymes and 1C consuming enzymes for all four models during differentiation (median of n = 3). (**b**) Log2 LFQ intensities of one-carbon cycle enzymes in primary cells (n = 7, two-sided paired Student’s t-tests, FDR < 0.05; error bars in the box plot spread from min to max; box extends from the 25th to 75th percentile; line represents median). (**c**) and (**d**) Immunofluorescence microscopy of ALDH1L1 and ALDH1L2 in hAPC preadipocytes and adipocytes, respectively. In the overlay, BODIPY is shown in grey, ALDH1L1 and ALDH1A2 in green, and TOM20 in magenta. Scale bar = 50 µm in overlay and 10 µm in inlay. Representative images of three experiments are shown. (**e**) Heatmap of log2 LFQ intensities for glycine transporters and glycine-degrading enzymes (median of n = 3). Source data

An additional unexpected finding was that the increase in mitochondrial protein mass during adipogenesis was accompanied by a decline in the levels of mitochondrial enzymes involved in one-carbon metabolism, a pathway that activates and transfers one-carbon units for biosynthetic processes (Fig. 4a). Similar to BCAA degradation, we observed for the one-carbon cycle an interplay of protein levels and protein localization to reorganize in a way that might enhance cytosolic nicotinamide adenine dinucleotide phosphate (NADPH) synthesis, required as a reduction equivalent to sustain lipid synthesis (Fig. 4d). While levels of all mitochondrial enzymes of the pathway decreased, the cytosolic branch of the pathway was upregulated in cell models and pAcs versus the SVF (Extended Data Fig. 8a,b). Meanwhile, both isoforms of 10-formyltetrahydrofolate dehydrogenase, ALDH1L1 and ALDH1L2, which catalyse the final reaction of the pathway and promote NADPH release, changed localization from the mitochondria to cytosolic protein complexes, as indicated by their protein profiles, as well as confirmed by co-immunostaining with the mitochondrial marker translocase of outer mitochondrial membrane 20 (TOM20) in SGBS cells and in hAPCs (Fig. 4e,h and Extended Data Fig. 8c,d). Cytosolic enzymes for purine and methionine synthesis-catalysing reactions consuming one-carbon metabolism intermediates and cytosolic NADPH decreased (Fig. 4d and Extended Data Fig. 8a). Notably, in proliferating cells, the electrochemical potential difference between mitochondria and the cytosol is responsible for driving the serine cycle in the direction that catabolizes serine in the mitochondria and synthesizes it in the cytosol, as shown in previous studies^35^; however, when the activity of the mitochondrial part of the serine cycle is reduced, the direction of the cytosolic part of the cycle is reversed, leading to cytosolic serine degradation and NADPH^36^ production. Given this regulatory mechanism, compartment-specific adjustments of enzyme levels during adipogenesis may also lead to an increase in cytosolic NADPH synthesis, thereby supporting de novo lipogenesis by providing the necessary reduction equivalents.

A predicted consequence of cytosolic serine degradation is glycine accumulation, as supported by metabolomic tracing studies that demonstrated an increase in glycine synthesis balanced by a decrease in glycine uptake in adipognesis^34^. In line with this, our data further showed a downregulation of glycine transporters while upregulating the glycine cleavage system, which is recognized as the primary enzymatic system responsible for glycine degradation induced by high glycine levels (Extended Data Fig. 8e).

Together, our findings highlight the coordinated control of protein localization and levels to reprogramme metabolic pathways to provide building blocks and reduction equivalents for fatty acid synthesis in adipogenesis.

### Spatial organization of lipid metabolism in human white adipocytes

**Fig. 5:**
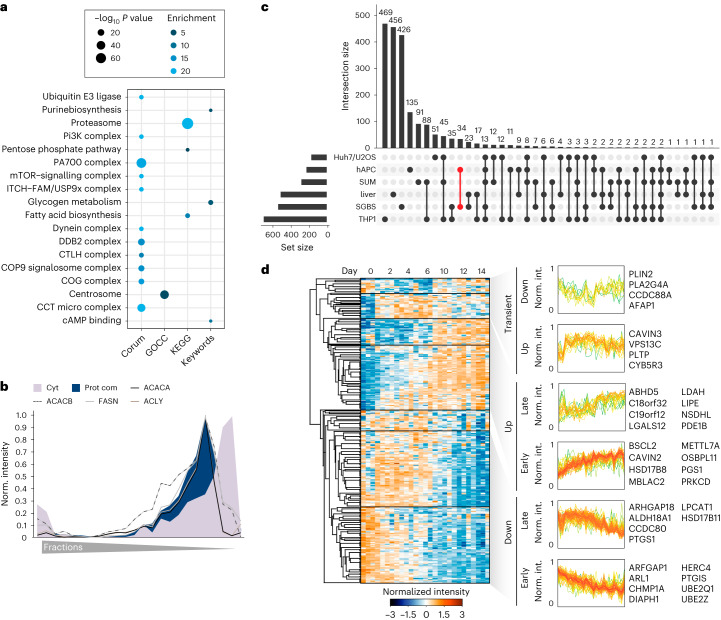
Spatial organization of lipid metabolism in adipocytes. **a**, KEGG, Keywords, CORUM and GO-term enrichment analyses of proteins identified in the protein complex cluster of PCP in adipocytes (one-sided Fisher’s exact test, enrichment score > 2, Benjamini–Hochberg FDR < 0.15). **b**, Profiles of enzymes involved in de novo fatty acid synthesis from citrate overlaid with a median profile of cytosolic and protein complex-associated proteins. **c**, Upset plot showing overlay of LD proteomes from SGBS and hAPC cells with LD proteomes from different cell lines and the liver from the LDKP. Set sizes of proteins are indicated in the bottom left bar graph and numbers of proteins for the indicated combinations are indicated in the top bar graph. The combination of adipocyte-specific LD proteins is indicated in red. **d**, Supervised hierarchical clustering of temporal profiles of significantly altered LD proteins during adipogenesis. Clusters with distinct temporal responses are indicated and examples of proteins found in these clusters are shown. (Proteins filtered for significantly changed proteins in at least three of the four models, FDR < 10^−2^). Source data

**Extended Data Fig. 9:**
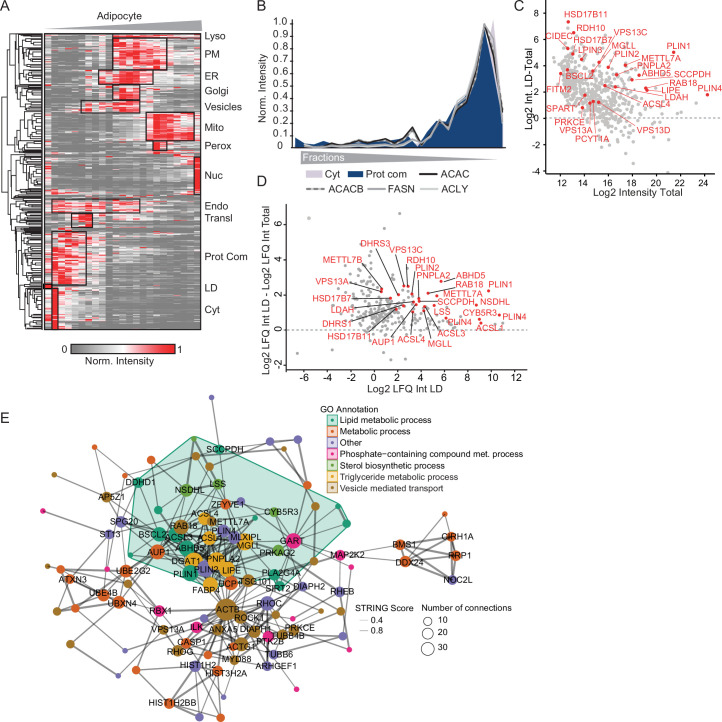
The LD proteome of human white adipocytes. (**a**) Supervised hierarchical clustering of temporal profiles of significantly altered LD proteins during adipogenesis. Hierarchical clustering of PCPs of hAPCs (n = 1). Enriched GO-terms in the clusters are indicated (Fisher’s test, FDR < 0.1). (**b**) Profiles of the indicated proteins involved in de novo lipogenesis in hAPCs overlaid with the marker profiles of cytosolic proteins and proteins from protein complexes. (**c**) and (**d**) Filtering of LD-assigned and LD-enriched proteins versus the total proteome. Protein levels of proteins determined as LD proteins as first or second assignments from SVMs based on PCP analysis in hAPCs in the LD fraction versus the total proteome from SGBS and hAPCs, respectively. (**e**) Functional clusters of LD proteins mapped in both adipocyte models were analysed using the STRING database, and those with at least two partners (physical and/or functional) are shown. High-confidence interactions with thicker connected lines. GO-enriched annotation terms (FDR < 5*10^(−2)) are coloured to highlight specific clusters. (Size = number of connections; line width = STRING combined score). Source data

The defining feature of white adipocytes is their specialization for lipid storage and the formation of large LDs. Therefore, we used our spatial-temporal atlas to investigate the organization of lipid metabolism and to define the adipocyte LD proteome. Hierarchical clustering of protein profiles revealed that proteins organized into protein complexes were clearly separated from cytosolic proteins using the PCP approach in SGBS cells (Fig. 2c). Unexpectedly, annotation enrichment analysis identified not only the partitioning of several prominent complexes, including mTOR, proteasome or chaperonin complexes, into this protein complex cluster, but also an enrichment for fatty acid biosynthesis (Fig. 5a). Notably, the enzymes ATP-citrate-lyase (ACLY), fatty acid synthase (FASN), acetyl-CoA carboxylase A (ACACA) and acetyl-CoA carboxylase B (ACACB), which catalyse the steps of fatty acid biosynthesis, were sorted into this cluster. Their protein profiles were nearly identical (Fig. 5b), suggesting potential condensate formation or a special arrangement of these enzymes within the cytosol in adipocytes. This co-fractionation of de novo lipogenesis enzymes with proteins forming complexes was reproduced in a PCP experiment in hAPCs (Extended Data Fig. 9a,b and Supplementary Table 5), thereby suggesting that the assembly of proteins involved in fatty acid synthesis into larger arrangements is a common feature of adipocytes.

Although adipocytes are the major cell type for lipid storage, a high confidence adipocyte LD proteome is lacking so far. To establish this and to exclude contaminants from the set of proteins with LD classifications, we selected for proteins that were enriched in the LD fraction compared with the total proteome, as all known LD marker proteins showed this behaviour (Extended Data Fig. 9c,d). Clustering analysis of significantly altered LD proteins mapped in adipocytes and present in at least two datasets of LD proteomes (Fig. 5c) revealed well-coordinated and time-dependent regulation of the LD proteome during adipogenesis, which exhibited high consistency across all the models examined (Fig. 5d). Following the induction of adipogenesis, a rapid surge in seipin (BSCL2) levels was observed. BSCL2 plays a crucial role in early LD formation by controlling the budding from the ER^37^. In contrast, several proteins displayed an upregulation pattern in the later stages. Among these, we identified several proteins functioning in lipid mobilization including hormone-sensitive lipase (LIPE) or ABHD5. The ubiquitously expressed member of the perilipin family, PLIN2, was transiently downregulated before levels peaked again in mature adipocytes. In addition, several proteins reported to function in lipid transfer and the regulation of inter-organelle contacts were induced or downregulated at certain time points of differentiation, indicating that these proteins might specifically modulate organelle contacts and lipid transfer during the differentiation process.

### C19orf12 is an adipocyte LD protein regulating lipid turnover

**Fig. 6:**
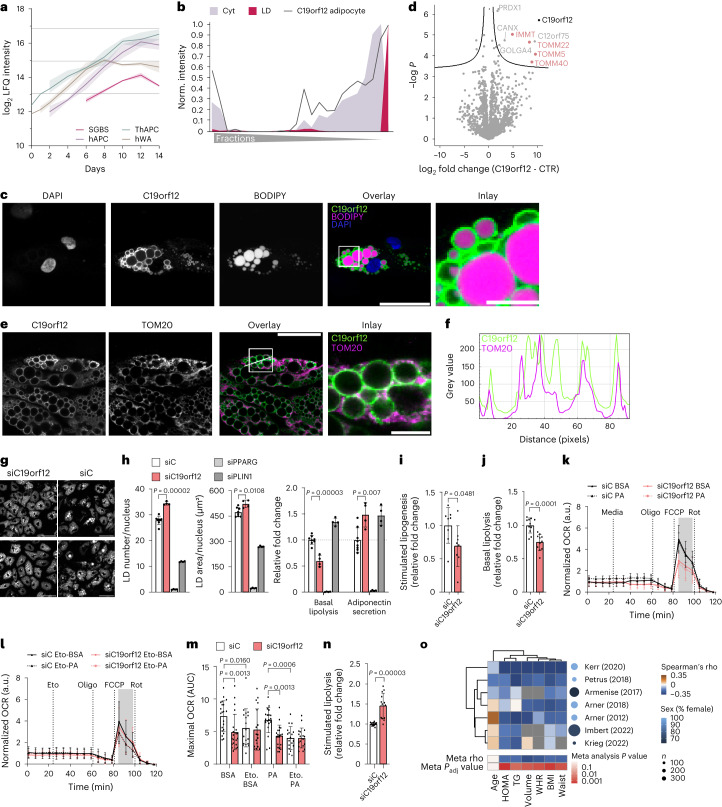
C19orf12 regulates adipocyte lipid turnover. **a**, Temporal regulation of C19orf12 levels during adipogenesis (lines represent mean and light areas entire range, *n* = 3). **b**, Protein profile of C19orf12 in SGBS cells overlaid with the indicated organelle marker profiles. **c**, Representative immunofluorescence of C19orf12 in SGBS adipocytes. Scale bars, 50 μm and 10 μm in the inlay. The experiment was repeated three times. **d**, Volcano plot of the interactome of C19orf12-GFP versus GFP control in SGBS preadipocytes overexpressing the GFP-tagged protein. The components of the mitochondrial protein import machinery are indicated in pink (*n* = 4, FDR < 0.05). **e**, Representative immunofluorescence of C19orf12 in SGBS adipocytes. Scale bars, 50 μm and 10 μm in the inlay. The experiment was repeated four times. **f**, Intensity plot of the fluorescence signals for C19orf21 and TOM20 on the line indicated in **e**. **g**, Two representative images of BODIPY staining in hAPCs on day 12 of differentiation after C19orf12 and control siRNA treatments 1 day before differentiation (*n* = 4, experiment was repeated twice). Scale bar, 80 μm. **h**, LD number, LD area, basal lipolysis and adiponectin secretion for hAPCs treated as in **g** (*n* = 8 siControl; *n* = 4 target siRNA, experiment was repeated twice, plot shows mean ± s.d., independent unpaired two-tailed *t*-test). **i**, Stimulated lipogenesis measurements in hAPCs on day 13 of differentiation after siRNA treatment on day 8 (*n* = 8, experiment repeated twice, plot shows mean ± s.d., independent unpaired two-tailed *t*-test). **j**,**n**, Basal and stimulated lipolysis in hAPCs on day 12 of differentiation after siRNA treatment on day 8 (*n* = 11 replicates, repeated twice for **j**, *n* = 13 replicates, repeated three times for **n**, plot shows mean ± s.d., independent unpaired two-tailed *t*-test). **k**–**m**, OCR measurement on day 12 of differentiation and its quantification in hAPCs treated with siRNA on day 8 in the presence of either BSA or palmitate and treated with etomoxir (*n* = 21 replicates, repeated three times for **k**; *n* = 18 replicates, repeated twice for etomoxir (Eto) in **l**, mean ± s.d., Kruskal–Wallis with uncorrected Dunn’s). a.u., arbitrary units; AUC, area under the curve. **o**, Association between C19orf12 expression and clinical parameters. BMI, body mass index; HOMA, homoeostatic model assessment of insulin sensitivity; TG, triglyceride; WHR, waist–hip ratio. Spearman rank correlation test was performed for the transcriptome analysis of WAT. Source data

**Extended Data Fig. 10:**
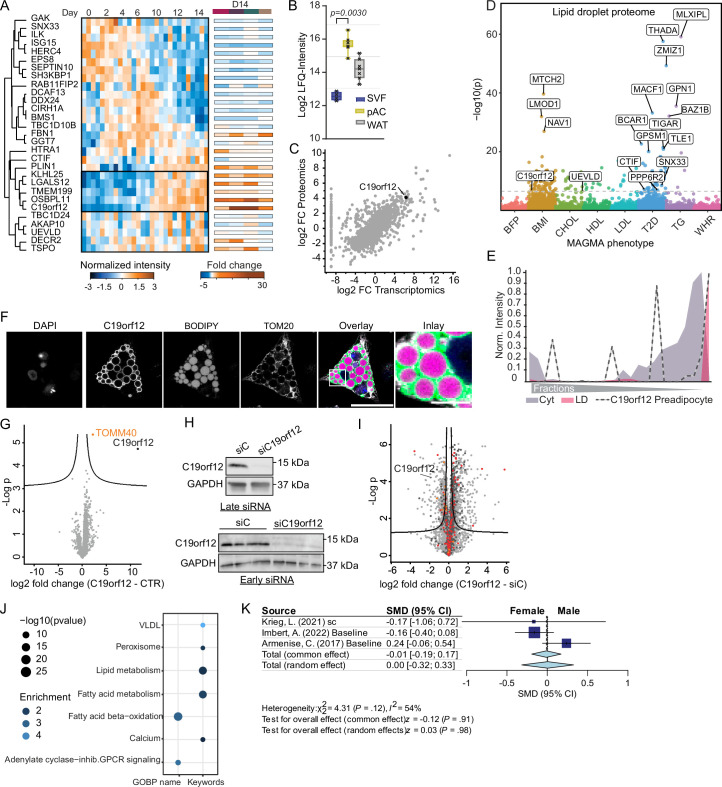
C19orf12 is a regulator of adipocyte function. (**a**) Supervised hierarchical clustering of z-scored temporal profiles of adipocyte LD proteins with altered levels. (**b**) C19orf12 levels in SVFs, pACs, and WAT (n = 7, two-sided paired Student’s t-tests, FDR < 0.05; error bars spread from min to max; box extends from the 25th to 75th percentile; line represents median). (**c**) Correlation of fold change at protein and mRNA levels between days 0 and 14. (**d**) Manhattan plot of metabolic associations (−log10 p values) in LD proteome. (**e**) Protein profile of C19orf12 in preadipocytes (**f**) Immunofluorescence of C19orf12 expression in hAPCs. BODIPY is shown in magenta, C19orf12 in green, TOM20 in grey, DAPI in blue. Scale bar = 50µm, 10µm in the inlay. Representative images of three experiments. (**g**) Volcano plot of C19orf12-GFP interactome in SGBS adipocytes. Mitochondrial protein import machinery indicated in pink (n = 4, two-sided Student’s t-test, FDR < 5*10(-2)). (**h**) Western blotting for C19orf12 in hAPCs treated with C19orf12 or control siRNA either 1 day before differentiation or at day 8 of differentiation (n = 1 for early knockdown, other replicates were subjected to proteomic analysis in (I)) and n = 1 in the late knockdown, n = 4). (**i**) Volcano plot of proteome analysis of early C19orf12 KD (n = 4, FDR < 0.05, S0 = 0.1). (**j**) Enrichment analysis for C19orf12 KD. Enrichment scores are indicated by bubble size and *P* values by colour code. (one-sided Fisher’s exact test, enrichment score >2, Benjamini-Hochberg FDR < 0,1) (**k**) Secondary meta-analysis for association between C19orf12 expression with clinical parameters (Fig. 6o) for sex specific effects. Forest plot indicating sex differences across cohorts were calculated as standardized mean difference (SMD), shown as blue squares, where the size correlates to the number of individuals in each cohor, lines represent 95% confidence interval. The total effect was calculated using either the common or random effects model depicted as light blue diamonds, width represents 95% confidence interval. No sex differences were observed (p = 0.91 and p = 0.98). Source data

To identify adipocyte LD proteins as potential candidates to promote the exceptional characteristics of adipocytes for lipid storage and dynamics, we overlaid the LD proteome from SGBS cells with the LD proteome from hAPCs. In the adipocyte LD proteomes, we detected most of the known LD proteins with many of these proteins involved in triglyceride and sterol metabolism (Extended Data Fig. 9e). More specifically, we identified 59 LD proteins that were common to both white adipocyte models, out of which 34 were exclusively mapped to LDs to adipocytes (Fig. 5c). These potential adipocyte-specific LD proteins were distinguished by their lack of enrichment in LDs in any of the datasets integrated into the LDKP^26^, which encompasses proteomic data from non-adipocyte cell lines and the liver. The integration of the core proteome data of adipogenesis with the adipocyte-specific LD proteome revealed 29 LD proteins that exhibited significant regulation during adipogenesis. Among these candidates, C19orf12 particularly captured our attention due to its conserved temporal trajectory across all four human adipogenesis models (Fig. 6a) and the substantial increase in mRNA expression and protein levels exceeding all other candidate proteins in the cell lines and pACs when compared with the SVFs, suggesting a potential functional role in adipocyte lipid storage (Extended Data Fig. 10a–c).

C19orf12 is a protein of unknown function associated with the neurodegenerative disease MPAN^9^. Previous studies reported various cellular localizations, including the cytosol, ER and mitochondria^38^, and a dysregulation in lipid metabolic genes upon loss of function^9^. Additionally, *C19orf12* is genetically associated with body mass index (Extended Data Fig. 10d). While *C19orf12* shows especially high expression in adipocytes versus other cell types^39^, the localization and function of the corresponding protein in fat cells remains unknown. Therefore, we first aimed to verify the subcellular distribution (Fig. 6b and Extended Data Fig. 10e) of C19orf12 predicted by PCP via immunofluorescence in SGBS and hAPCs. In both cell types, we observed C19orf12 immunostaining around the LDs (Fig. 6c and Extended Data Fig. 10f).

To gain insight into the C19orf12 interactome on LDs, we conducted co-immunoprecipitation (co-IP) coupled with proteomics to identify binding proteins in preadipocytes and mature adipocytes. In addition to the uncharacterized protein, C12orf75, we identified interactions between C19orf12 and proteins involved in protein folding and the Golgi apparatus protein GOLGA4, as well as several proteins involved in mitochondrial protein import. Specifically, we pulled down TOM40, the channel-forming subunit of the TOM complex, TOM22 and TOM5 (Fig. 6d and Extended Data Fig. 10g). While a minor Golgi apparatus localized pool, which was also visible in the PCP profile (Fig. 6b), might interact with Golgin subfamily A member 4 (GOLGA4), the interaction with the TOM complex indicates that the main pool of C19orf12 binds concurrently to LDs and the mitochondrial import machinery, thereby functioning at LD–mitochondrial interaction sites. Co-immunostaining for C19orf12 and the mitochondrial marker TOM20 confirmed the colocalization of C19orf12 with mitochondria in close proximity to LDs (Fig. 6e,f).

Next, we sought to explore the functional importance of C19orf12 for adipocyte differentiation and lipid storage. For this, we performed knockdowns in both hAPC preadipocytes and differentiated adipocytes. Early gene silencing resulted in a sustained decrease in C19orf12 protein levels throughout the differentiation process (Extended Data Fig. 10h). Proteomic characterization of the C19orf12 knockdown cells on day six of adipogenesis uncovered an upregulation of mitochondrial, peroxisomal, fatty acid synthesis and degradation proteins (Extended Data Fig. 10i). These proteome changes were also reflected in altered lipid dynamics in the mature adipocytes. More specifically, we found a higher number of LDs and a twofold reduction in basal lipolysis following knockdown of *C19orf12* (Fig. 6g,h). At the same time, *C19orf12*-depleted cells secreted ~50% more adiponectin than cells transfected with control short-interfering RNA (siRNA; Fig. 6h). These findings suggest a role for C19orf12 in adipocyte differentiation, as well as an impact on lipid metabolism as indicated by the reduction in basal lipolysis upon C19orf12 knockdown (Fig. 6h).

Consequently, to study the role of C19orf12 in lipid metabolism in differentiated adipocytes, we depleted this protein at day 8 post-adipogenic induction (Extended Data Fig. 10h). Compared with control cells, C19orf12 silencing resulted in a reduction of insulin-stimulated lipid synthesis and basal lipolysis, suggesting impaired lipid turnover (Fig. 6i,j). To test whether this was linked to alterations in lipid utilization, we performed bioenergetic analyses using Seahorse. Our data revealed that C19orf12 depletion resulted in a reduction in maximal oxygen consumption rates (OCRs) (Fig. 6k,m). This could either be due to a reduction in (1) the action of lipases on the LDs, (2) lipid utilization in mitochondria or (3) a general impairment in mitochondrial function. To distinguish between these possibilities, we first assessed the maximal lipolytic capacity. We found that maximal isoprenaline-induced lipolysis was increased in C19orf12-depleted versus control cells, suggesting that lipases can still function on the LDs (Fig. 6n). Next, to assess whether the effects were dependent on fatty acid levels we incubated the cells with exogenous palmitate. As displayed in Fig. 6k,m, we found that this did not affect the reduction in OCR following C19orf12 silencing, suggesting that fatty acid abundance is not a limiting factor. In addition, we performed bioenergetic assays in the presence of etomoxir, a CPT1 inhibitor that prevents fatty acid entry into the mitochondria (Fig. 6l,m). We observed that C19orf12 knockdown and control siRNA cells displayed similar OCR. This indicates that utilization of other energy substrates (for example, carbohydrates and amino acids) is intact and that there is no general impairment in mitochondrial function. Altogether, these results together with the organellar localization and interactions with the TOM complex, indicate that C19orf12 determines adipocyte lipid turnover by affecting the capability of mitochondria to metabolize long-chain fatty acids.

Finally, to define a possible clinical relevance of our results, we analysed the relationship between C19orf12 mRNA levels and fat cell parameters in human WAT. Transcriptomic profiles in several independent cohorts^40–46^ revealed an inverse correlation between *C19orf12* expression and body fat, adipocyte cell volume, plasma triglyceride levels and insulin resistance (measured by the homoeostatic model assessment of insulin resistance), which was not affected by sex (Fig. 6o and Extended Data Fig. 10k). In brief, our results indicate that C19orf12 is an adipocyte LD–mitochondrial contact site protein with a crucial function in determining lipid turnover in adipocytes.

## Discussion

Here, we integrate temporal proteomic profiling with a spatial PCP approach to map cellular rearrangements throughout adipogenesis. Our findings underscore the importance of alterations in both protein abundance and localization during adipocyte differentiation. In contrast to the use of 3T3L1 murine adipocytes in most studies, we utilized proteomic profiles from four distinct human adipogenesis models. This allowed us to reveal the consistent temporal trajectory of adipogenesis in human cells unaffected by immortalization procedures, differentiation protocols and donor-specific effects.

While previous investigations have predominantly focused on changes in the proteome or transcriptome during cellular differentiation, the impact of protein localization has largely remained unexplored. Our research indicates that, beyond the 38% of quantified proteins changing levels, an additional 20% undergo translocation during adipogenesis. This suggests a crucial role for protein translocation in cellular differentiation. We posit that shifts in protein localization may play a yet underappreciated role in the determination of cell fate and specialization processes beyond adipogenesis.

In the context of adipogenesis, we hypothesize that numerous protein localization events play a key role in priming adipocytes for lipid storage and regulate multiple cellular functions in parallel. These events may facilitate the transition from a state of high proliferation to a mature state characterized by high mitochondrial and lipid content. One instance is the reorganization of the translational machinery. During adipogenesis, cells cease dividing and decrease their demand for overall protein synthesis, while simultaneously increasing the production of mitochondrial proteins. We observed a reduction in proteins involved in general protein synthesis in our proteomic time course, along with changes in the composition of the translational machinery, which may favour proper synthesis of mitochondrial proteins, as we see recruitment of the NATC, important for properly modifying mitochondrial proteins^31^. Another example for how differentiating adipocytes optimize their spatial organization for lipid synthesis is the concurrent upregulation of lipogenic enzymes and their potential spatial organization into larger assemblies, as indicated by nearly identical proteomic fractionation profiles. Indeed, phase separation and condensate formation are frequently employed mechanisms that govern the biochemical activities of enzymes and maintain metabolic homoeostasis and a recent study the formation of ACACA-citrate dimers promoted polymerization into larger structures with increased enzymatic activity^47^. Our study supports this finding and indicates the formation of even more complex condensates involving all enzymes throughout de novo lipogenesis, potentially facilitating efficient substrate channelling and regulating substrate flux along this pathway.

Our study has uncovered a reprogramming of various additional metabolic pathways during adipogenesis. Specifically, we found that protein levels and localization are regulated in concert to reprogramme BCAA catabolism and one-carbon metabolism, probably to provide building blocks and reduction equivalents for de novo lipogenesis. We suggest that BCAA catabolism is redirected from the cytosol in preadipocytes, where it fuels nucleotide biosynthesis to a new trajectory, leading to BCAA degradation and synthesis of citrate and acetyl-CoA in mitochondria. This reallocation is facilitated by the upregulation of all mitochondrial enzymes in the pathway and the translocation of BCAT2 from the cytosol to the mitochondria. Indeed, previous studies have demonstrated that BCAT2 assembles with downstream mitochondrial enzymes into a metabolome complex and may enhance flux through the mitochondrial portion of the pathway^48^.

Another pathway where protein levels and localization coordinately drive reprogramming towards support of de novo lipogenesis is one-carbon metabolism. We have identified counter regulation and localization changes of enzymes involved in the mitochondrial and the cytosolic branch of the cycle. This coordinated regulation may result in the redirection of the cytosolic part of the cycle towards serine degradation and NADPH production, similar to that reported for the ablation of mitochondrial one-carbon metabolism enzymes^36^. This rearrangement may contribute to increase cytosolic NADPH pools necessary for providing reduction equivalents for fatty acid synthesis. Our findings at the proteomics level are in line with results from a recent state-of-the-art metabolomic tracing study comparing the metabolism of preadipocytes and mature adipocytes^34^. The findings support the remodelling of BCAA catabolism, revealing that 30% of lipogenic acetyl-CoA pools originate from BCAA catabolism. Furthermore, they demonstrate an increase in serine uptake and synthesis in mature adipocytes. Together with our data, these observations suggest a potential effort by the cell to increase its serine pool, directing it towards NADPH synthesis.

Finally, our spatiotemporal atlas of adipogenesis has revealed several unknown factors that may be crucial for adipocyte function. For example, we identify C19orf12 as an important protein determining lipid turnover in adipocytes. In C19orf12-depleted cells, we observed impaired mitochondrial utilization of lipids, whereas metabolism of other fuel sources remained unchanged. This alteration in lipid metabolism is further exhibited by a reduction in basal lipolysis, a potential compensatory response to the inability to metabolize released lipids, as increased stimulated lipolysis indicates that lipases are present and activatable. Alternatively, previous research has proposed a feedback regulatory mechanism in lipolysis triggered by the accumulation of fatty acids^49^, which may explain the concurrent increase in lipid accumulation and stimulated lipogenesis observed in our study.

How C19orf12 functions at the molecular level and impacts adipocyte lipid turnover remains unknown. The simultaneous interaction of the protein with LDs and mitochondria, specifically with the TOM complex involved in mitochondrial protein import, presents various potential explanations for impacting lipid turnover and mitochondrial fatty acid utilization. For instance, by interacting with TOM40, it may regulate protein composition and thereby the functions of LD-associated mitochondria, given the documented distinct proteomic characteristics of mitochondria associated with LDs^50^. Alternatively, C19orf12 could directly play a role in regulating the transfer of fatty acids to mitochondria. Notably, the TOM complex has been previously documented to localize at organelle contact sites^51^ and recent work proposes a function in lipid transfer^52^. Our findings on C19orf12 depletion diverge from those of another adipocyte LD–mitochondrial contact site protein, mitoguardin-2 (MIGA2). While knockdown of C19orf12 enhanced lipid accumulation and reduced basal lipolysis, the knockdown of MIGA2 results in decreased adipogenesis and lipid accumulation^53^. These distinct outcomes further imply the existence of diverse types of organelle contacts within adipocytes, playing possibly distinct roles in either promoting lipogenesis or facilitating lipid degradation. Of note, C19orf12 expression is inversely correlated with factors related to adiposity and insulin resistance in human cohorts, validating the relevance of our in vitro knockdown experiments and emphasizing the clinical importance of C19orf12 in human metabolism.

In summary, we address a major gap in our current understanding of human adipogenesis, revealing cellular and proteomic changes that occur during the formation of fat cells. By analysing temporal proteomics data from multiple models of human adipogenesis and incorporating spatial proteomics, we have uncovered a highly coordinated process of cellular remodelling. Our findings offer a high-resolution view of the sequential changes in protein isoforms, abundance and organelle organization, elucidating the spatial organization of metabolic processes in adipogenesis. Through generating a comprehensive cellular map of human adipocytes, we have developed a resource that offers researchers investigating LDs, adipogenesis and adipocyte function a platform to analyse protein expression, metabolic pathways and organelle composition throughout adipogenesis.

There are some limitations of our study. Similar to other organelle-profiling techniques such as localization of organelle proteins by isotope tagging^54^ and differential organellar maps^16^, our study’s method, PCP, relies on distinctive fractionation behaviours to map protein localization. In our study, PCP has demonstrated the capability to map not only organelles but also membrane-less compartments and protein complexes; however, it is crucial to recognize the inherent limitations of PCP, particularly its inability in resolving protein complexes that are associated with organelles. Moreover, the possibility of co-fractionation due to close organelle interactions, as occurred for peroxisomes and the ER in adipocyte precursors in our study, may lead to co-fractionation and similar abundance patterns, potentially causing misinterpretations of protein localization, which needs to be avoided through strict quality control measures.

## Methods

### Human sample acquisition

Samples from subcutaneous abdominal WAT were obtained by needle aspiration under local anaesthesia (as described elsewhere^4^) from five women and two men (mean ± s.d. for age 60.7 ± 3.5 years and body mass index 28.7 ± 6.6 kg m^−2^) (Supplementary Table 3). Mature fat cells and SVF were isolated by collagenase digestion as previously described^12^. All studies were approved by the Regional Board of Ethics in Stockholm. All participants provided informed written consent and sex was self-reported. A small compensation for the potential discomfort was given to participants undergoing fine needle biopsies according to Swedish guidelines and the approved ethical permits.

### Cell culture

Human preadipocytes were cultured and differentiated according to their respective protocols^10,12,14^. Cells were cultured from frozen stocks and culture was performed in a humidified atmosphere with 5% CO_2_ at 37 °C. Differentiation schemes and origins are specified in Supplementary Tables 1 and 2. The detailed proteomic characterization of the cell models in this study supported their authentication as human adipocytes.

### RNA isolation, cDNA synthesis and real-time qPCR

Total RNA was purified using the NucleoSpin RNA kit (740955, Macherey-Nagel). Concentration and purity were measured using a NanoDrop 2000 spectrophotometer (Thermo Fisher Scientific). Reverse transcription and mRNA measurements were performed with iScript cDNA synthesis (1708891, Bio-Rad) and iQ SYBR Green Supermix (1708882, Bio-Rad) kits, respectively. Relative mRNA levels were calculated with the comparative Ct-method: 2^DCt-target gene^/2^DCt-reference gene^. The following primers were used: *PLIN1* (fwd, TGGAGACTGAGGAGAACAAG; rev, ATGTCACAGCCGAGATGG); *LIPE* (HSL; fwd, AGCCTTCTGGAACATCACCG; rev, ATCTCAAAGGCTTCGGGTGG); *CEBPA* (fwd, AGCCTTGTTTGTACTGTATG; rev, AAAATGGTGGTTTAGCAGAG); and *PPARG* (fwd, CCCAGAAAGCGATTCCTTCAC; rev, AGCTGATCCCAAAGTTGGTGG). We used *18s* (fwd, TGACTCAACACGGGAAACC; rev, TCGCTCCACCAACTAAGAAC) as a housekeeping gene.

### siRNA knockout

For knockdown in proliferating hAPCs, siRNAs were introduced via reverse transfection using DharmaFECT (T-2003-04, Dharmacon) before differentiation, as previously described^55^. Late knockdown was performed through electroporation using the Neon System (Invitrogen) on day 8 of differentiation, as previously described^56^. An siGenome SMARTpool siRNA, comprising a mixture of four siRNA, was used to target C19orf12 (Dharmacon, M-014731-01), while siRNA targeting PLIN1 (Dharmacon, M-019595-01) and PPARG (Dharmacon, M-003436-02) were used as positive controls for adipogenesis. siGENOME non-targeting control siRNA pool 1 (Dharmacon, D-001206-13-05) was used as a negative control.

### Immunofluorescence

Cells were seeded on glass plates and fixed with 4% paraformaldehyde (SC281692, Santa Cruz Biotechnology), permeabilized with 0.1% Triton X-100 for 10 min and blocked with 1% BSA in PBS for 1 h. Overnight incubation with primary antibodies (SLC25A10, 1:100 dilution (Atlas Antibodies, HPA023048, lot A105751), TOMM20, 1:500 dilution (Thermo Fisher, H00009804, lot L1181-4F3), ALDH1L1, 1:100 dilution (Atlas Antibodies, HPA050139, lot 000016054), ALDH1L2, 1:100 dilution (Atlas Antibodies, HPA039481, lot A106389), C19orf12, 1:500 dilution (Atlas Antibodies, HPA046930, lot 000019650) and BCAT2, 1:100 dilution (Proteintech, 16417-1-AP, lot 00089129)) at 4 °C was followed by PBS washes and 1 h secondary antibody incubation (goat anti-rabbit coupled to AF-647, 1:500 dilution (Thermo Fisher Scientific, A27040, lot SA245805), goat anti-mouse IgG (H + L) Cross-Adsorbed Secondary Antibody Cy3, 1:500 dilution (Thermo Fisher Scientific, A10521, lot 2017376)). Cells were stained with BODIPY 493/503 and 4,6-diamidino-2-phenylindole (DAPI; Invitrogen, R37606) for 10 min. Imaging was performed using a Leica TCS SP8 microscope with the LasX software (Leica, v.1.4.4) with a ×63 glycerol objective, at 100 nm per pixel resolution. Fiji software^57^ was used for image analysis, with images being median projections of three confocal sections after background subtraction.

### Adiponectin assay

For the analysis of adiponectin secretion, the cell culture medium was exchanged on day 12 of differentiation and collected on day 14. Adiponectin levels were determined by ELISA (R&D systems, DRP300) according to the manufacturer’s protocol.

### Basal and induced lipolysis

Cells were plated in 96-well plates (10386612, Fisher Scientific). On day 10, the cell culture medium was exchanged to 100 μl phenol red-free DMEM/F12 (21041-033, Thermo Scientific), supplemented according to Supplementary Table 2. On day 12, the cell culture medium was collected for basal lipolysis and exchanged to 80 µl phenol red-free DMEM/F12 supplemented with 2% fatty acid free BSA (3117057001, Sigma-Aldrich) and 0.1 mg ml^−1^ ascorbic acid (500074, Merck), with or without 1 µM isoproterenol (I5627-5G, Sigma-Aldrich) for 3 h, after which the medium was collected. Glycerol release into the culture medium was quantified to measure lipolysis^55^. Then, 20 µl medium or glycerol standards (G7793-5ML, Sigma-Aldrich) was transferred to a 96-well plate (M5686-40EA, Sigma-Aldrich). A 100 µl mixture of Free Glycerol Reagent (F6428-40ML, Sigma-Aldrich) and Amplex Ultrared (10737474, Fisher Scientific) was added and incubated for 15 min at room temperature before measurement in a Varioskan microplate reader (Thermo Fisher Scientific) at Excitation/Emission 530/590 nm. Glycerol release was normalized to cell number.

### Quantification of LD number and area

Cells were cultured in 96-well plates (10386612, Fisher Scientific). On day 12 of differentiation, cells were fixed in PBS with 4% paraformaldehyde (SC281692, Santa Cruz Biotechnology) for 10 min and washed with PBS. LDs and nuclei were stained with BODIPY 493/503 (1:2,500 dilution, D3922, Thermo Fisher Scientific) and Hoechst (1:5,000 dilution, ABCAAB228551, VWR) for 10 min. Scanning was performed using CellInsight CX5 (Thermo Fisher Scientific) and quantified by employing object (nuclei) and spot (LD) detection algorithm in HCS Studio software (Thermo Fisher Scientific). LD area and number were normalized to nuclei count.

### Beta-oxidation

Cells were plated in 96-well plates (102416-100, Agilent). Beta-oxidation was measured using the Agilent Seahorse XF Palmitate Oxidation Stress Test kit (103693-100, Agilent) according to the manufacturer’s instructions. Wave and Wave Desktop (Agilent Technologies, v.2.6.3.5) were used to acquire and analyse Seahorse experiments. On day 11 of differentiation, the cell medium was changed to substrate-limited growth medium, consisting of Seahorse XF DMEM medium (103575-100) supplemented with 0.5 mM glucose, 1 mM glutamine and 0.5 mM l-carnitine, along with standard D11 medium (Supplementary Table 1). On day 12, the medium was changed to Seahorse XF DMEM supplemented with 2 mM glucose and 0.5 mM l-carnitine and incubated at 37 °C in a non-CO_2_ incubator for 1 h. Before the assay, the medium was replaced with 150 µl fresh medium and 30 µl either BSA or palmitate-BSA was added per well. A palmitate oxidation stress test was performed using the XF96 Seahorse Extracellular Flux Analyzer (Agilent) to measure OCR. Then, 4 μM etomoxir (inhibitor of mitochondrial uptake of long-chain fatty acids and beta-oxidation), 1.5 μM oligomycin (inhibitor of ATP synthesis), 1.5 μM carbonyl cyanide-p-trifluoromethoxyphenylhydrazone (FCCP; uncoupling agent) and 0.5 μM rotenone/antimycin A (inhibitors of complex I and complex III) were injected sequentially. Oxygen consumption was normalized using CyQUANT (Thermo Fisher) according to the manufacturer’s instructions and corrected for non-mitochondrial respiration (obtained after addition of rotenone/antimycin A). The area under the curve for maximal respiration (after addition of FCCP) was calculated. As normality was not met, a nonparametric test was run for statistical analysis.

### Co-IP

SGBS cells were electroporated using the Neon Transfection System (Invitrogen) with C-terminally tagged *C19orf12* or GFP control. The plasmid was cloned from Origene rc231802 into the pegfp-N1 Gateway destination vector (gifted from R. Shaw, supplied by Addgene, plasmid #31796). Two days after transfection, cells were washed with ice-cold PBS and scraped in ice-cold Co-IP buffer (10 mM Tris/HCl pH 7.5, 150 mM NaCl, 0.5 mM EDTA and protein inhibitor cocktail (Roche)) supplemented with 0.5% Nonident P40. Lysates were incubated on ice for 30 min and cleared by centrifugation (4 °C, 10 min, 17,000*g*). The supernatant was diluted using 3× the volume of Co-IP buffer without detergent and incubated with washed anti-GFP magnetic agarose, 1:20 dilution (ChromoTek, gtma-20, lot 90122001MA)) for 1 h at 4 °C. Beads were washed with Co-IP buffer + 0.05% NP40 and Co-IP buffer without detergent. For the digest, beads were first incubated with 50 µl elution buffer I (2 M urea, 50 mM Tris/HCl, pH 7.5, 20 µg µl^−1^ Trypsin (Sigma, t6567) and 1 mM dithiothreitol) for 30 min at 37 °C, 1,300 rpm. Afterwards, beads were incubated in 50 µl elution buffer II (2 M urea, 50 mM Tris/HCl, pH 7.5 and 5 mM chloroacetamide) in the dark. Supernatants were digested, combined overnight at 25 °C, 1,000 rpm and peptides were acidified using 1 µl trifluoroacetic acid and purified on C18 Stage Tips.

### Western blot

Cells were lysed in RIPA buffer (89901, Thermo Fisher Scientific) supplemented with protease (11836170001, Merck) and phosphatase (4906837001, Millipore) inhibitors, centrifuged at 15,000*g* for 10 min and heated at 50 °C for 5 min in Laemmli buffer (1610747, Bio-Rad). Proteins were separated by SDS–PAGE and transferred to PVDF membranes as previously described^56^. Antibodies against C19orf12 (1:500 dilution, Proteintech, 27382-1-AP), GAPDH (1:1,000 dilution, Cell Signaling Technology, 2118) and HRP linked rabbit IgG (1:10,000 dilution, Cell Signaling Technology, 7074S) were used.

### Organelle fractionation

PCP was performed as previously described^23^. For differentiated SGBS and hAPC cells, 5 × 150-mm dishes of cells at day 20 after differentiation, and for preadipocyte gradients, 3× T175 flasks of SGBS cells 1 day after confluency, were used per replicate. Cells were homogenized with a tissue homogenizer on ice in a 1:1 mixture of scraped cells to lysis buffer (20% sucrose, 20 mM Tris, pH 7.4, 0.5 mM EDTA, 5 mM KCl, 3 mM MgCl_2_, protease inhibitor and phosphatase inhibitor cocktail (Roche)). Then, 2 ml supernatant was loaded onto a continuous 11 ml 20–55% sucrose gradient in 20 mM Tris, pH 7.4, 0.5 mM EDTA, 5 mM KCl and 3 mM MgCl_2_. Organelles were separated at 100,000*g* (Beckmann, Rotor SW40 Ti) for 3 h at 4 °C. To isolate LDs, the 1-ml top fraction was cut with a tube-slicer (Beckman Coulter). The underlying 0.5-ml fractions were collected from the top to the bottom of the gradient.

### Proteomics

#### Sample preparation

Cells were washed with ice-cold PBS, scraped, boiled for 5 min at 95 °C and 1,000 rpm in 2% SDC buffer (2% SDC and 100 mM Tris-HCl, pH 8.5) and sonicated (Diagenode Bioruptor, 15 × 30 s). Protein concentration was determined via BCA Protein Assay (Thermo, 23225). After overnight digestion (37 °C, 1,000 rpm) with a 1:50 ratio (protein:enzyme) of trypsin (Sigma, t6567) and LysC (Wako, 129-02541), proteins were reduced and alkylated with 10 mM TCEP and 40 mM chloroacetamide at 40 °C in the dark for 10 min. Peptides were acidified by adding 1:1 (v:v) of isopropanol and 2% TFA. After centrifugation for 10 min at 15,000*g*, supernatants were loaded onto activated triple-layer styrenedivinylbenzene reversed-phase sulfonated Stage Tips (3M Empore). Peptides were washed with 100 µl ethylacetate 1% TFA, 100 µl 30% methanol 1% TFA and 150 µl 0.2% TFA and eluted with 60 µl elution buffer (80% ACN and 5% NH_4_OH). Peptides were lyophilized and dissolved in 10 µl MS loading buffer (2% ACN and 0.1% TFA).

#### LC–MS/MS

LC–MS/MS analysis of 500 ng peptides was performed on a Orbitrap Exploris 480 (Thermo Fisher Scientific) equipped with a nano-electrospray ion source and FAIMS (CV50) coupled with an EASY-nLC 1200 HPLC (all Thermo Fisher Scientific). Peptides were separated at 60 °C on 50-cm columns with an inner diameter of 75 μm packed in-house with ReproSil-Pur C18-AQ 1.9 μm resin (Dr. Maisch) over 1 h by reversed-phase chromatography using a binary buffer system consisting of buffer A (0.1 formic acid) and buffer B (80% ACN and 0.1% formic acid). Starting with 5% of buffer B, this fraction was increased stepwise to 45% over 45 min followed by a washout at 95%, at a constant flow rate of 300 nl min^−1^. Peptides were ionized and transferred from the LC system into to the gas phase using electrospray ionization. A DIA tandem mass spectrometry 1 h method was used. One MS1 scan (300–1,650 m/z, maximum ion fill time of 45 ms, normalized AGC target of 300%, *R* = 120,000 at 200 m/z) was followed by 66 MS2 fragment scans of unequally spaced windows (fill time of 22 ms, normalized AGC target of 1,000%, normalized HCD collision energy of 30%, *R* = 15,000). Spectra were acquired in profile mode using positive polarity.

### Bioinformatics

#### Statistics and reproducibility

Due to the nature of cell culture experiments, sample randomization was not possible. As most studies were performed by individual researchers who were aware of the design of the experiments, blinding during data collection and analysis was not performed. No statistical methods were used to predetermine sample sizes, but our sample sizes are similar to those reported in previous publications^56^. For statistical analysis of all cellular experiments, normality and equal variances were formally tested using the D’Agostino–Pearson omnibus test. If the data did not follow normal (Gaussian) distribution, nonparametric analysis was performed as identified in the relevant Methods section. Analysis of the assays was performed using Microsoft Excel 2016 and Prism (GraphPad Software, v.9.5.1). Figures were created with Adobe Illustrator and BioRender.

#### Proteomic data processing

DIA raw files were analysed using Spectronaut software (v.15.7.220308.50606 and v.15.7.220308.50606, Copernicus, developed by Biognosys) with directDIA and searched against the Uniprot human databases: UP000005640_9606 and UP000005640_9606_additional with standard processing parameters (trypsin cleavage with a peptide length ranging from 7 to 52 amino acids, two missed cleavages). Fixed modification settings included carbamidomethylation and variable modifications were methionine oxidation and N-terminal acetylation. The analysis specified a minimum of three and a maximum of six Best N Fragment ions per peptide. For filtering and quality control, a precursor and protein *q* value cutoff of 1% was applied. A global normalization of data based on median quantities was implemented to correct for any MS intensity drift over time. To apply the proteomic ruler^19^, settings were changed as previously published^58^.

#### Proteome analysis

Data processing was conducted in Perseus (v.1.6.15.0) using default settings, except where noted. Second localization assignments for PCP were generated with Perseus (v.1.5.6.2). Proteomics quality control was performed in Spectronaut. UMAP was generated using the UMAP package (v.0.5.3) in Python (v.3.8.8); Circular plots were generated with Circos Table Viewer^59^. Enrichment analyses and Manhattan plots were visualized using ggplot package in the R statistical computing environment v.4.0.2.R. Before analysis, contaminants and reverse decoy database hits were excluded. Data distribution was assumed to be normal but this was not formally tested. No statistical methods were used to predetermine sample sizes, but our sample sizes are similar to those reported in previous publications^23,24^. Reported intensities were calculated using the median value between replicates, only considering conditions with at least two quantifications.

For proteome analysis, label-free quantitation (LFQ) values were log_2_-transformed and proteins were filtered for at least two valid values in at least one condition. Data were width-adjusted and missing values were imputed from a normal distribution with a downshift of 1.8 and a width of 0.3. Significantly regulated proteins were determined by ANOVA (permutation-based FDR < 0.01) or for two conditions by two-sided Student’s *t*-test (permutation-based FDR < 0.01). No samples were excluded from analysis for the time course experiment and one sample was excluded from the analysis in Extended Data Fig. 1 as it did not pass quality control. For Fisher’s exact tests, annotations were extracted from UniProtKB, Gene Ontology (GO), the Kyoto Encyclopedia of Genes and Genomes (KEGG) and CORUM. Complete enrichment analyses are available in Source Data and representative categories are visualized. Supervised Euclidian hierarchical clustering was performed on *z*-scored temporal profiles of common time points of all cell models. Time points not present across all models were excluded from the correlation analysis. For each *z*-scored temporal protein profile pairwise Pearson correlations between the cell models were calculated and proteins with positive correlations in all combinations were defined to have a conserved temporal trajectory.

For protein copy number analysis, the proteomic ruler plugin^19^ for the Perseus software was used. Copy numbers were filtered for at least two valid values per condition. Missing values were imputed from a normal distribution with a downshift of 1.8 and a width of 0.3. Significantly regulated proteins between the time points were determined by ANOVA (permutation-based FDR < 0.01). Ranked copy numbers of the significant outliers were *z*-scored before hierarchical clustering. Pairwise correlations between the cell models were calculated and proteins with positive correlations in all combinations were defined to have a conserved temporal trajectory.

#### Integration of proteome and transcriptome

For the integration of proteome and transcriptome, the proteomic time course from hAPCs of the temporally conserved core proteome was matched with a previously generated hAPC transcriptome during differentiation^21^. Spearman’s rho correlations between transcriptome normalized and log_2_-transformed count data and proteome log_2_-transformed LFQ values were calculated, as well as between the *z*-scored temporal profiles of transcriptome and proteome for time points present in both datasets. One-dimensional annotation enrichment was performed on Pearson correlation values of the temporal profiles.

#### PCP analysis

LFQ intensities for each protein among the organellar fractions were scaled from 0–1. For the generation of median protein, the median values from the biological replicates for each fraction were calculated and a second 0–1 scaling step was performed. Pearson correlations between profiles of biological replicates were calculated and proteins with Pearson correlation <0 in any of the comparisons, were excluded.

For SVM-based organelle predictions, a marker list was compiled from experimentally validated localizations by Itzahk et al.^24^ and the Protein Atlas^27^, with LD markers supplemented from the LDKP^26^. The marker set was used for parameter optimization (Sigma of 0.2 and *C* = 8) and training of the SVMs with RBF Kernel implemented into the Perseus software. Four replicates (for SGBS mature adipocytes), three replicates (for preadipocytes) and one replicate (hAPC cells) were concatenated for organelle classifications using Perseus’s second organelle assignment option. The algorithm estimates secondary subcellular compartment contributions by identifying the highest Pearson correlation between the experimental protein profiles and in silico combinations of median organelle profiles, generated by incremental mixing of the profile of the first organelle assignment with other organelle markers.

Organelle predictions were filtered for positive assignment to at least one organelle. The *α* value is a quantitative measure for the second organelle contribution. Proteins with an *α* value of 0 were considered specific to a single organelle. The prediction accuracy for marker proteins was calculated based on the correct prediction of markers for each compartment. Proteins were defined as having a localization change if the first organelle assignments were different.

### Human genetic association analysis

Human genetic association analysis was performed using MAGMA (multi-marker analysis of genomic annotation)^60^ scores in the Type2 diabetes Knowledge Portal^61^. MAGMA scores for genes of LD proteins were plotted for adiposity-related phenotypes. A significance threshold of *P* < 2.5 × 10^−6^ is generally considered significant for MAGMA. A more stringent significance threshold of *P* < 3.125 × 10^−7^ was derived by Bonferroni correction to account for the eight phenotypes tested.

### C19orf12 clinical correlations

The expression level of C19orf12 was retrieved from previously published WAT microarray and RNA sequencing studies consisting of a total of 784 participants with or without obesity, of which 171 (21.8%) were male and 613 (78.2%) were female^40–46^. Data are publicly available in the NCBI Gene Expression Omnibus repository under accession nos. GSE25401, GSE199063, GSE141221, GSE95640, GSE59034 and GSE113080. In brief, C19orf12 expression was correlated to clinical parameters using the rcorr function in the Hmisc v.5.0-1 package in R (method = ‘spearman’) for each cohort, after which correlation and *n* values were analysed in a meta-analysis using the metacor function in the meta v.6.2-1 R package. Resulting meta rho and *P* values across all cohorts were extracted and plotted using pheatmap v.1.0.12 and ggplot2 v.3.4.2 (clinical correlation) or visualized as a forest plot using the meta package (sex differences). The standardized mean difference was calculated by dividing the mean difference by the s.d. and plotted for each cohort with sufficient numbers of females and males.

### Reporting summary

Further information on research design is available in the Nature Portfolio Reporting Summary linked to this article.

### Supplementary information

Reporting Summary

Supplementary Table 1 Summary of human adipogenesis models used in the study.

Supplementary Table 2 Information on individual differentiation protocols.

Supplementary Table 3 Information on human donors.

Supplementary Table 4 Proteomic profiles during adipogenesis.

Supplementary Table 5 PCP in SGBS and hAPC.

### Source data

Source Data Fig. 1 Statistical Source Data Fig. 1.

Source Data Fig. 2 Statistical Source Data Fig. 2.

Source Data Fig. 3 Statistical Source Data Fig. 3.

Source Data Fig. 4 Statistical Source Data Fig. 4.

Source Data Fig. 5 Statistical Source Data Fig. 5.

Source Data Fig. 6 Statistical Source Data Fig. 6.

Source Data Extended Data Fig.1 Statistical Source Data Extended Data Fig. 1.

Source Data Extended Data Fig. 2 Statistical Source Data Extended Data Fig. 2.

Source Data Extended Data Fig. 3 Statistical Source Data Extended Data Fig. 3.

Source Data Extended Data Fig. 4 Statistical Source Data Extended Data Fig. 4.

Source Data Extended Data Fig. 5 Statistical Source Data Extended Data Fig. 5.

Source Data Extended Data Fig. 6 Statistical Source Data Extended Data Fig. 6.

Source Data Extended Data Fig. 7 Statistical Source Data Extended Data Fig. 7.

Source Data Extended Data Fig. 8 Statistical Source Data Extended Data Fig. 8.

Source Data Extended Data Fig. 9 Statistical Source Data Extended Data Fig. 9.

Source Data Extended Data Fig. 10 Statistical Source Data Extended Data Fig. 10.

Source Data Extended Data Fig. 10 Unprocessed western blots Extended Data Fig. 10.

## Data Availability

Proteomic raw data and Spectronaut search tables are available via ProteomeXchange under identifier PXD047412. Proteomic data are also provided as Supplementary Tables. Transcriptomics data are available from the DNA Data Bank of Japan under accession nos. DRA000991, DRA002711, DRA002747 and DRA002748. Microcopy raw data are available on Figshare at 10.6084/m9.figshare.25267462, 10.6084/m9.figshare.25273423 and 10.6084/m9.figshare.25267462 (refs. ^62,63^). Source data are provided with this paper.

## Code availability

No custom code was used in the study.
