# Supplementary material for: A spatiotemporal proteomic map of human adipogenesis
Source: Nat Metab. 2024 Apr 2;6(5):861–79. doi: 10.1038/s42255-024-01025-8 (PMC11132986; doi:10.1038/s42255-024-01025-8)
Supplement: Supplementary file 1 — Reporting Summary [file 42255_2024_1025_MOESM1_ESM.pdf]

nature portfolio

Corresponding author(s): Natalie Krahmer

Last updated by author(s): Feb 19, 2024

Reporting Summary

Nature Portfolio wishes to improve the reproducibility of the work that we publish. This form provides structure for consistency and transparency in reporting. For further information on Nature Portfolio policies, see our Editorial Policies and the Editorial Policy Checklist.

Statistics

For all statistical analyses, confirm that the following items are present in the figure legend, table legend, main text, or Methods section.

n/a	Confirmed	
☐	☒	The exact sample size (*n*) for each experimental group/condition, given as a discrete number and unit of measurement
☐	☒	A statement on whether measurements were taken from distinct samples or whether the same sample was measured repeatedly
☐	☒	The statistical test(s) used AND whether they are one- or two-sided<br>*Only common tests should be described solely by name; describe more complex techniques in the Methods section.*
☐	☒	A description of all covariates tested
☐	☒	A description of any assumptions or corrections, such as tests of normality and adjustment for multiple comparisons
☐	☒	A full description of the statistical parameters including central tendency (e.g. means) or other basic estimates (e.g. regression coefficient) AND variation (e.g. standard deviation) or associated estimates of uncertainty (e.g. confidence intervals)
☐	☒	For null hypothesis testing, the test statistic (e.g. *F*, *t*, *r*) with confidence intervals, effect sizes, degrees of freedom and *P* value noted<br>*Give P values as exact values whenever suitable.*
☒	☐	For Bayesian analysis, information on the choice of priors and Markov chain Monte Carlo settings
☒	☐	For hierarchical and complex designs, identification of the appropriate level for tests and full reporting of outcomes
☐	☒	Estimates of effect sizes (e.g. Cohen's *d*, Pearson's *r*), indicating how they were calculated

Our web collection on statistics for biologists contains articles on many of the points above.

Software and code

Policy information about availability of computer code

Data collection	Leica TCS SP8 Confocal Laser Scanning Microscope uses LasX software (Leica, v1.4.4); TiE microscope (Nikon) was used together with its NIS Elements software; lipid droplet imaging and quantification on the CX5 microscope was performed with HCS Studio software (Thermo Fisher Scientific)
Data analysis	Raw proteomics data was processed with Spectronaut (v15.7.220308.50606, v18.4.231017.55695 for revision Analysis of proteomic ruler, early adipogenesis and method evaluation experiments), Imputation and statistical analysis was performed in Perseus (v.1.6.15.0), except for second localization assignments generated with Perseus (1.5.6.2). For Protein copy number estimation, proteomic ruler plugin v1.6.2 was used in Perseus. Analysis of assays and summarizing protein intensities was performed using Microsoft Excel 2016 and Prism (Graphpad Software LLC, v9.5.1). For image analysis the Fiji distribution of ImageJ was utilized. R (R v4.1.2) was used for transcriptomic analysis; UMAPS were generated using the umap package (v0.5.3) in Python (v3.8.8); Wave and Wave Desktop (Agilent Technologies, v.2.6.3.5) were used to acquire and analyze Seahorse experiments; Manhattan plots were generated in R using ggplot2; Circular plots were generated with Circos Table Viewer(https://doi.org/10.1101/gr.092759.109)

For manuscripts utilizing custom algorithms or software that are central to the research but not yet described in published literature, software must be made available to editors and reviewers. We strongly encourage code deposition in a community repository (e.g. GitHub). See the Nature Portfolio guidelines for submitting code & software for further information.

Data

Policy information about availability of data

All manuscripts must include a data availability statement. This statement should provide the following information, where applicable:

- Accession codes, unique identifiers, or web links for publicly available datasets
- A description of any restrictions on data availability
- For clinical datasets or third party data, please ensure that the statement adheres to our policy

Proteomic raw data and Spectronaut search tables are available via ProteomeXchange under the identifier:
PXD047412 . No password protection.
Previously published and reanalyzed WAT microarray and RNA sequencing studies are publicly available in the NCBI Gene Expression Omnibus repository under the accession numbers GSE25401, GSE199063, GSE141221, GSE95640, GSE59034 and GSE113080
Microcopy raw data are available at Figshare numbers 10.6084/m9.figshare.25267462, 10.6084/m9.figshare.25273423, 10.6084/m9.figshare.25267462.5640

Research involving human participants, their data, or biological material

Policy information about studies with human participants or human data. See also policy information about sex, gender (identity/presentation), and sexual orientation and race, ethnicity and racism.

Reporting on sex and gender	Sex was self reported by participants for biopsies and the information is summarized in Supplementary Table3. In each case, consent was obtained for collecting and sharing information about sex. No analysis segregated for sex specific effects was performed since the number of male participants (n=2) was too low. Biological sex of cell lines as determined in their original publications is reported in Supplementary Table 1. Cell line proteomics were analyzed according to their biological sex in Extended data figures 3H and 3I. Human WAT transcriptomics data have been previously reported on. In each case, consent was obtained for collecting and sharing information about sex. Transcriptomics analysis of publicly available data sets of both men and women were used to examine whether correlations between C19orf12 expression and BMI were dependent on gender (Extended Data Fig.10K)
Reporting on race, ethnicity, or other socially relevant groupings	To our knowledge, information regarding race, ethnicity or other relevant groupings was not reported in the publications of the analyzed cell lines. No similar information is available on donors of primary samples.
Population characteristics	Primary samples of five women and two men (mean± standard deviation for age 60.7±3.5 years and body mass index 28.7 ±6.6 kg/m2) were used. Sex and age and genotypic information of cell line donors is summarized in Supplementary Table1.
Recruitment	Generation and analysis of new data was done retrospectively and no new subjects were recruited for this publication.
Ethics oversight	The study was performed in agreement with the Declaration of Helsinki and approved by the Stockholm regional board of Ethics. All patients provided written informed consent before participation.A small compensation was given to participants undergoing fine needle biopsies according to Swedish guidelines and the approved ethical permits.

Note that full information on the approval of the study protocol must also be provided in the manuscript.

Field-specific reporting

Please select the one below that is the best fit for your research. If you are not sure, read the appropriate sections before making your selection.

☒ Life sciences ☐ Behavioural & social sciences ☐ Ecological, evolutionary & environmental sciences

For a reference copy of the document with all sections, see nature.com/documents/nr-reporting-summary-flat.pdf

Life sciences study design

All studies must disclose on these points even when the disclosure is negative.

Sample size	No sample size calculation was performed for in vitro analysis, sample sizes were determined based on our experience from previous studies. Proteomics time course: While matching or exceeding replicates (n=3) of similar studies (https://doi.org/10.15252/msb.20209813, https://doi.org/10.1016/j.celrep.2022.110604) our study focused on the inclusion of different models (n=4), observation of consistent trends and verification using clinical isolates. Number of clinical isolates (n=7) was determined by availability and practicality but proved to be sufficent for statistical analysis. Proteomic gradients: In line with previous studies provided by our and other groups (https://doi.org/10.1016/j.devcel.2018.09.017, https://doi.org/10.1038/s41467-018-08191-w). Adipocyte functional assays: Matched or exceeded number of replicates. (https://doi.org/10.1016/j.cmet.2017.07.012, https://doi.org/10.1007/s00125-013-2954-5).
Data exclusions	Time point day8 of the SGBS differentiation was lost during sample preparation and was subsequently omitted from the study design. Early proteomics 66 window method time point 24h replicate 3(of 4) was excluded due to a skewed quantification profile. First two attempts to show C19orf12 knockdown on western blot did not show the expected bands due to aggregate formation during boiling of the sample, solved by RT incubation. Second replicate of the Seahorse experiment was excluded due to stunted oligomycin response, potentially due to

suboptimal cell density.

Replication	Functional readouts were repeated a minimum of two times to ensure reproducibility (indicated in manuscript). See above for exclusions. Microscopy shows representative images from at least three experiments.
Randomization	No randomization was performed. Consistent quality of proteomic measurements was ensured by observing protein number, PCA, CoV (Extended Data Fig.1) and other parameters (chromatograms, methionine oxidation, digestion efficiency etc.). Shifts in data quality would be more likely to be observed there than in single protein trends that are consistent across biological models.
Blinding	Since most studies were performed by individual researchers knowing the design of the studies, blinding during data collection and analysis was not performed.

Behavioural & social sciences study design

All studies must disclose on these points even when the disclosure is negative.

Study description	*Briefly describe the study type including whether data are quantitative, qualitative, or mixed-methods (e.g. qualitative cross-sectional, quantitative experimental, mixed-methods case study).*
Research sample	*State the research sample (e.g. Harvard university undergraduates, villagers in rural India) and provide relevant demographic information (e.g. age, sex) and indicate whether the sample is representative. Provide a rationale for the study sample chosen. For studies involving existing datasets, please describe the dataset and source.*
Sampling strategy	*Describe the sampling procedure (e.g. random, snowball, stratified, convenience). Describe the statistical methods that were used to predetermine sample size OR if no sample-size calculation was performed, describe how sample sizes were chosen and provide a rationale for why these sample sizes are sufficient. For qualitative data, please indicate whether data saturation was considered, and what criteria were used to decide that no further sampling was needed.*
Data collection	*Provide details about the data collection procedure, including the instruments or devices used to record the data (e.g. pen and paper, computer, eye tracker, video or audio equipment) whether anyone was present besides the participant(s) and the researcher, and whether the researcher was blind to experimental condition and/or the study hypothesis during data collection.*
Timing	*Indicate the start and stop dates of data collection. If there is a gap between collection periods, state the dates for each sample cohort.*
Data exclusions	*If no data were excluded from the analyses, state so OR if data were excluded, provide the exact number of exclusions and the rationale behind them, indicating whether exclusion criteria were pre-established.*
Non-participation	*State how many participants dropped out/declined participation and the reason(s) given OR provide response rate OR state that no participants dropped out/declined participation.*
Randomization	*If participants were not allocated into experimental groups, state so OR describe how participants were allocated to groups, and if allocation was not random, describe how covariates were controlled.*

Ecological, evolutionary & environmental sciences study design

All studies must disclose on these points even when the disclosure is negative.

Study description	*Briefly describe the study. For quantitative data include treatment factors and interactions, design structure (e.g. factorial, nested, hierarchical), nature and number of experimental units and replicates.*
Research sample	*Describe the research sample (e.g. a group of tagged Passer domesticus, all Stenocereus thurberi within Organ Pipe Cactus National Monument), and provide a rationale for the sample choice. When relevant, describe the organism taxa, source, sex, age range and any manipulations. State what population the sample is meant to represent when applicable. For studies involving existing datasets, describe the data and its source.*
Sampling strategy	*Note the sampling procedure. Describe the statistical methods that were used to predetermine sample size OR if no sample-size calculation was performed, describe how sample sizes were chosen and provide a rationale for why these sample sizes are sufficient.*
Data collection	*Describe the data collection procedure, including who recorded the data and how.*
Timing and spatial scale	*Indicate the start and stop dates of data collection, noting the frequency and periodicity of sampling and providing a rationale for these choices. If there is a gap between collection periods, state the dates for each sample cohort. Specify the spatial scale from which the data are taken*
Data exclusions	*If no data were excluded from the analyses, state so OR if data were excluded, describe the exclusions and the rationale behind them, indicating whether exclusion criteria were pre-established.*
Reproducibility	*Describe the measures taken to verify the reproducibility of experimental findings. For each experiment, note whether any attempts to repeat the experiment failed OR state that all attempts to repeat the experiment were successful.*

Randomization

Describe how samples/organisms/participants were allocated into groups. If allocation was not random, describe how covariates were controlled. If this is not relevant to your study, explain why.

Blinding

Describe the extent of blinding used during data acquisition and analysis. If blinding was not possible, describe why OR explain why blinding was not relevant to your study.

Did the study involve field work? ☐ Yes ☒ No

Field work, collection and transport

Field conditions

Describe the study conditions for field work, providing relevant parameters (e.g. temperature, rainfall).

Location

State the location of the sampling or experiment, providing relevant parameters (e.g. latitude and longitude, elevation, water depth).

Access & import/export

Describe the efforts you have made to access habitats and to collect and import/export your samples in a responsible manner and in compliance with local, national and international laws, noting any permits that were obtained (give the name of the issuing authority, the date of issue, and any identifying information).

Disturbance

Describe any disturbance caused by the study and how it was minimized.

Reporting for specific materials, systems and methods

We require information from authors about some types of materials, experimental systems and methods used in many studies. Here, indicate whether each material, system or method listed is relevant to your study. If you are not sure if a list item applies to your research, read the appropriate section before selecting a response.

Materials & experimental systems

n/a	Involved in the study	
☐	☒	Antibodies
☐	☒	Eukaryotic cell lines
☒	☐	Palaeontology and archaeology
☒	☐	Animals and other organisms
☐	☒	Clinical data
☒	☐	Dual use research of concern
☒	☐	Plants

Methods

n/a	Involved in the study	
☒	☐	ChIP-seq
☒	☐	Flow cytometry
☒	☐	MRI-based neuroimaging

Antibodies

Antibodies used

SLC25A10 (Atlas Antibodies, HPA023048, Lot: A105751), TOMM20 (Thermo Fisher, H00009804, Lot: L1181-4F3), ALDH1L1 (Atlas Antibodies, HPA050139, Lot: 000016054), ALDH1L2 (Atlas Antibodies, HPA039481, Lot: A106389), C19orf12 (Atlas Antibodies, HPA046930, Lot: 000019650), C19orf12 #2 (Proteintech, 27382-1-AP; Lot: 00053485), BCAT2 (Proteintech, 16417-1-AP, Lot:00089129), Goat anti-rabbit coupled to AF-647 (Thermo Fisher Scientific, A27040, LotSA245805), Goat anti-Mouse IgG (H+L) Cross-Adsorbed Secondary Antibody Cy3 (Thermo Fisher Scientific, A10521, Lot:2017376), immunoprecipitation: anti-GFP magnetic agarose (ChromoTek GmbH, gtma-20, Lot: 90122001MA); Western blotting: Anti rabbit IgG, HRP linked Antibody (7074S, Cell Signaling technologies), GAPDH (#2118, Cell Signaling)

Validation

SLC25A10 antibody: Vendor showed exclusive interaction with stated antigen via protein array, and mitochondrial localization via IF; used in previous publications for western blotting (10.1261/rna.076422.120); Our study: Localization in pre-/adipocytes agrees with spatial proteomics experiments.
TOMM20: Vendor showed IF consistent with mitochondrial localization, western blots showing the expected protein size, and dose dependent signal in ELISA format. Used for mitochondrial IF in publications (e.g.: DOI: 10.1126/science.abq4835); Shows distinct mitochondrial structures in our studies (esp. preadipocytes) and agrees with other mitochondrial targets (ALDH1L2)
ALDH1L1: Vendor shows agreeing IHC signal to second independent antibody for the same target and agreement with RNA seq signals across tissues, Interaction with own antigen in protein array, Western blots confirmed with capture MS (agreeing with antigen) and consistent with MS TMT data of the same sources; IF of vendor agrees with previously published information and our IF results in adipocytes.
ALDH2L2: Vendor provides IF and IHC data , protein array data showing single peak for interaction with own antigen and western blot showing the expected protein size; Used in other publications, e.g. for IF confirming mitochondrial localization (https://doi.org/10.1016/j.celrep.2023.112562). Our study: IF agrees with spatial proteomics.
C19orf12: HPA046930: Vendor provides IF and IHC data , protein array data showing single peak for interaction with own antigen. IF localization in our study was confirmed using second cell line and agreed with localization using spatial proteomics and the second antibody for the same target (IF data not shown). 27382-1-AP: Vendor provides WB and IF evidence. Our study: shows correct size and validation via knock-down. Independent publication showing IF and WB: (PMID: 35182730).

BCAT2: Vendor provides WB evidence showing single band in different lysates; Independent publications showing WB and IF (https://doi.org/10.1038/s42255-021-00520-6, 10.3390/antiox11030583)

Eukaryotic cell lines

Policy information about cell lines and Sex and Gender in Research

Cell line source(s)	Compare Supplementary Table1<br>Adipogenesis models were originally isolated from subcutaneous white adipose tissue of different donors. SGBS (male) were originally reported in Wabitsch et al., 2001 and kindly provided by Prof. Dr. med. Martin Wabitsch (Department of Paediatrics and Adolescent Medicine, Ulm University Medical Centre); hAPC (male) were first reported in Ehrlund et al., 2013, Karolinska Institutet) ; ThAPC cells were derived from hAPCs by immortalization at the Karolinska institute (Couchet, M. et al., 2023). Both hAPC and ThAPC were provided by Dr. Niklas Mejhert and Dr. Mikael Ryden (Department of Medicine, Karolinska Institutet). The hWA model (female) was isolated and immortalized as reported in Markussen et al., 2017 and provided by Prof. Dr. Jacob Hansen (Department of Biology, University of Copenhagen, Copenhagen, Denmark)
Authentication	Cell lines were authenticated by detailed proteomic characterization in this study.
Mycoplasma contamination	Cell lines tested negative for mycoplasma (PanReac AppliChem by ITW Reagents, A3744, Lot: 2107145)
Commonly misidentified lines<br>(See ICLAC register)	No commonly misidentified cell lines were used.

Palaeontology and Archaeology

Specimen provenance	*Provide provenance information for specimens and describe permits that were obtained for the work (including the name of the issuing authority, the date of issue, and any identifying information). Permits should encompass collection and, where applicable, export.*
Specimen deposition	*Indicate where the specimens have been deposited to permit free access by other researchers.*
Dating methods	*If new dates are provided, describe how they were obtained (e.g. collection, storage, sample pretreatment and measurement), where they were obtained (i.e. lab name), the calibration program and the protocol for quality assurance OR state that no new dates are provided.*

☐ Tick this box to confirm that the raw and calibrated dates are available in the paper or in Supplementary Information.

Ethics oversight	*Identify the organization(s) that approved or provided guidance on the study protocol, OR state that no ethical approval or guidance was required and explain why not.*

Note that full information on the approval of the study protocol must also be provided in the manuscript.

Animals and other research organisms

Policy information about studies involving animals; ARRIVE guidelines recommended for reporting animal research, and Sex and Gender in Research

Laboratory animals	*For laboratory animals, report species, strain and age OR state that the study did not involve laboratory animals.*
Wild animals	*Provide details on animals observed in or captured in the field; report species and age where possible. Describe how animals were caught and transported and what happened to captive animals after the study (if killed, explain why and describe method; if released, say where and when) OR state that the study did not involve wild animals.*
Reporting on sex	*Indicate if findings apply to only one sex; describe whether sex was considered in study design, methods used for assigning sex. Provide data disaggregated for sex where this information has been collected in the source data as appropriate; provide overall numbers in this Reporting Summary. Please state if this information has not been collected. Report sex-based analyses where performed, justify reasons for lack of sex-based analysis.*
Field-collected samples	*For laboratory work with field-collected samples, describe all relevant parameters such as housing, maintenance, temperature, photoperiod and end-of-experiment protocol OR state that the study did not involve samples collected from the field.*
Ethics oversight	*Identify the organization(s) that approved or provided guidance on the study protocol, OR state that no ethical approval or guidance was required and explain why not.*

Note that full information on the approval of the study protocol must also be provided in the manuscript.

Clinical data

Policy information about clinical studies

All manuscripts should comply with the ICMJE guidelines for publication of clinical research and a completed CONSORT checklist must be included with all submissions.

Clinical trial registration	N/A
Study protocol	N/A
Data collection	N/A
Outcomes	N/A

Dual use research of concern

Policy information about dual use research of concern

Hazards

Could the accidental, deliberate or reckless misuse of agents or technologies generated in the work, or the application of information presented in the manuscript, pose a threat to:

No	Yes	
☒	☐	Public health
☒	☐	National security
☒	☐	Crops and/or livestock
☒	☐	Ecosystems
☒	☐	Any other significant area

Experiments of concern

Does the work involve any of these experiments of concern:

No	Yes	
☒	☐	Demonstrate how to render a vaccine ineffective
☒	☐	Confer resistance to therapeutically useful antibiotics or antiviral agents
☒	☐	Enhance the virulence of a pathogen or render a nonpathogen virulent
☒	☐	Increase transmissibility of a pathogen
☒	☐	Alter the host range of a pathogen
☒	☐	Enable evasion of diagnostic/detection modalities
☒	☐	Enable the weaponization of a biological agent or toxin
☒	☐	Any other potentially harmful combination of experiments and agents

Plants

Seed stocks	*Report on the source of all seed stocks or other plant material used. If applicable, state the seed stock centre and catalogue number. If plant specimens were collected from the field, describe the collection location, date and sampling procedures.*
Novel plant genotypes	*Describe the methods by which all novel plant genotypes were produced. This includes those generated by transgenic approaches, gene editing, chemical/radiation-based mutagenesis and hybridization. For transgenic lines, describe the transformation method, the number of independent lines analyzed and the generation upon which experiments were performed. For gene-edited lines, describe the editor used, the endogenous sequence targeted for editing, the targeting guide RNA sequence (if applicable) and how the editor was applied.*
Authentication	*Describe any authentication procedures for each seed stock used or novel genotype generated. Describe any experiments used to assess the effect of a mutation and, where applicable, how potential secondary effects (e.g. second site T-DNA insertions, mosiacism, off-target gene editing) were examined.*

ChIP-seq

Data deposition

☐ Confirm that both raw and final processed data have been deposited in a public database such as GEO.

☐ Confirm that you have deposited or provided access to graph files (e.g. BED files) for the called peaks.

Data access links<br>*May remain private before publication.*	*For "Initial submission" or "Revised version" documents, provide reviewer access links. For your "Final submission" document, provide a link to the deposited data.*
Files in database submission	*Provide a list of all files available in the database submission.*
Genome browser session<br>(e.g. UCSC)	*Provide a link to an anonymized genome browser session for "Initial submission" and "Revised version" documents only, to enable peer review. Write "no longer applicable" for "Final submission" documents.*

Methodology

Replicates	*Describe the experimental replicates, specifying number, type and replicate agreement.*
Sequencing depth	*Describe the sequencing depth for each experiment, providing the total number of reads, uniquely mapped reads, length of reads and whether they were paired- or single-end.*
Antibodies	*Describe the antibodies used for the ChIP-seq experiments; as applicable, provide supplier name, catalog number, clone name, and lot number.*
Peak calling parameters	*Specify the command line program and parameters used for read mapping and peak calling, including the ChIP, control and index files used.*
Data quality	*Describe the methods used to ensure data quality in full detail, including how many peaks are at FDR 5% and above 5-fold enrichment.*
Software	*Describe the software used to collect and analyze the ChIP-seq data. For custom code that has been deposited into a community repository, provide accession details.*

Flow Cytometry

Plots

Confirm that:

☐ The axis labels state the marker and fluorochrome used (e.g. CD4-FITC).

☐ The axis scales are clearly visible. Include numbers along axes only for bottom left plot of group (a 'group' is an analysis of identical markers).

☐ All plots are contour plots with outliers or pseudocolor plots.

☐ A numerical value for number of cells or percentage (with statistics) is provided.

Methodology

Sample preparation	*Describe the sample preparation, detailing the biological source of the cells and any tissue processing steps used.*
Instrument	*Identify the instrument used for data collection, specifying make and model number.*
Software	*Describe the software used to collect and analyze the flow cytometry data. For custom code that has been deposited into a community repository, provide accession details.*
Cell population abundance	*Describe the abundance of the relevant cell populations within post-sort fractions, providing details on the purity of the samples and how it was determined.*
Gating strategy	*Describe the gating strategy used for all relevant experiments, specifying the preliminary FSC/SSC gates of the starting cell population, indicating where boundaries between "positive" and "negative" staining cell populations are defined.*

☐ Tick this box to confirm that a figure exemplifying the gating strategy is provided in the Supplementary Information.

Magnetic resonance imaging

Experimental design

Design type	*Indicate task or resting state; event-related or block design.*

Design specifications | *Specify the number of blocks, trials or experimental units per session and/or subject, and specify the length of each trial or block (if trials are blocked) and interval between trials.*

Behavioral performance measures | *State number and/or type of variables recorded (e.g. correct button press, response time) and what statistics were used to establish that the subjects were performing the task as expected (e.g. mean, range, and/or standard deviation across subjects).*

Acquisition

Imaging type(s) | *Specify: functional, structural, diffusion, perfusion.*

Field strength | *Specify in Tesla*

Sequence & imaging parameters | *Specify the pulse sequence type (gradient echo, spin echo, etc.), imaging type (EPI, spiral, etc.), field of view, matrix size, slice thickness, orientation and TE/TR/flip angle.*

Area of acquisition | *State whether a whole brain scan was used OR define the area of acquisition, describing how the region was determined.*

Diffusion MRI ☐ Used ☐ Not used

Preprocessing

Preprocessing software | *Provide detail on software version and revision number and on specific parameters (model/functions, brain extraction, segmentation, smoothing kernel size, etc.).*

Normalization | *If data were normalized/standardized, describe the approach(es): specify linear or non-linear and define image types used for transformation OR indicate that data were not normalized and explain rationale for lack of normalization.*

Normalization template | *Describe the template used for normalization/transformation, specifying subject space or group standardized space (e.g. original Talairach, MNI305, ICBM152) OR indicate that the data were not normalized.*

Noise and artifact removal | *Describe your procedure(s) for artifact and structured noise removal, specifying motion parameters, tissue signals and physiological signals (heart rate, respiration).*

Volume censoring | *Define your software and/or method and criteria for volume censoring, and state the extent of such censoring.*

Statistical modeling & inference

Model type and settings | *Specify type (mass univariate, multivariate, RSA, predictive, etc.) and describe essential details of the model at the first and second levels (e.g. fixed, random or mixed effects; drift or auto-correlation).*

Effect(s) tested | *Define precise effect in terms of the task or stimulus conditions instead of psychological concepts and indicate whether ANOVA or factorial designs were used.*

Specify type of analysis: ☐ Whole brain ☐ ROI-based ☐ Both

Statistic type for inference | *Specify voxel-wise or cluster-wise and report all relevant parameters for cluster-wise methods.*
(See Eklund et al. 2016)

Correction | *Describe the type of correction and how it is obtained for multiple comparisons (e.g. FWE, FDR, permutation or Monte Carlo).*

Models & analysis

n/a	Involved in the study
☐	☐ Functional and/or effective connectivity
☐	☐ Graph analysis
☐	☐ Multivariate modeling or predictive analysis

Functional and/or effective connectivity | *Report the measures of dependence used and the model details (e.g. Pearson correlation, partial correlation, mutual information).*

Graph analysis | *Report the dependent variable and connectivity measure, specifying weighted graph or binarized graph, subject- or group-level, and the global and/or node summaries used (e.g. clustering coefficient, efficiency, etc.).*

Multivariate modeling and predictive analysis | *Specify independent variables, features extraction and dimension reduction, model, training and evaluation metrics.*
