# Supplementary material for: A spatiotemporal proteomic map of human adipogenesis
Source: Nat Metab. 2024 Apr 2;6(5):861–79. doi: 10.1038/s42255-024-01025-8 (PMC11132986; doi:10.1038/s42255-024-01025-8)
Supplement: Supplementary file 2 — Summary of human adipogenesis models used in the study. [file 42255_2024_1025_MOESM2_ESM.pdf]

Model	Sex	Isolation site	Donor	Immortalized	References
**SGBS**	m	sc	4-months old, SGBS syndrome	no	Wabitsch et al., 2001
**hAPC**	m	sc	16-y, healthy BMI: 24	no	Ehrlund et al., 2013
**ThAPC**	m	sc	16-y, healthy BMI: 24	TERT	Couchet et al., 2023
**hWA**	f	sc	48-y, BMI: 19.9	TERT	Markussen et al. 2017
