# Supplementary material for: A spatiotemporal proteomic map of human adipogenesis
Source: Nat Metab. 2024 Apr 2;6(5):861–79. doi: 10.1038/s42255-024-01025-8 (PMC11132986; doi:10.1038/s42255-024-01025-8)
Supplement: Supplementary file 3 — Information on individual differentiation protocols. [file 42255_2024_1025_MOESM3_ESM.pdf]

SGBS

			Day 1-4	Day 5-14
Insulin	0.02	µM		
Cortisol	100	nM		
T3	0.2	nM		
Transferrin	10	µg/ml		
IBMX	250	µM		
Rosiglitazone	2	µM		
Dexamethasone	25	nM		

hAPC/ ThAPC

			Day 1-3	Day 4-10	Day 11-14
Insulin	0.86	µM			
T3	0.2	nM			
Transferrin	10	µg/ml			
Rosiglitazone	0.2	µM			
IBMX	100	µM			
Dexamethasone	1000	nM			

hWA

			Day 1-6	Day 7-14
Insulin	0.86	µM		
Cortisol	1000	nM		
T3	1	nM		
Rosiglitazone	1	µM		
IBMX	500	µM		
Dexamethasone	1000	nM		
FBS	2	%		
