# Supplementary material for: A spatiotemporal proteomic map of human adipogenesis
Source: Nat Metab. 2024 Apr 2;6(5):861–79. doi: 10.1038/s42255-024-01025-8 (PMC11132986; doi:10.1038/s42255-024-01025-8)
Supplement: Supplementary file 4 — Information on human donors. [file 42255_2024_1025_MOESM4_ESM.pdf]

Publication id	gender	age	BMI	WHR	body fat %DXA	Disease	HbA1c
P1	Male	56	42.1	1.202	49.3	Obesity, hypertension	40
P2	Female	60	29.4	0.96	49.8	Obesity	44
P3	Female	61	24.9	0.842	36.5	Hyperlipidemia	43
P4	Female	63	24.5	0.931	41.2	Healthy	39
P5	Female	58	23.4	0.89	40.4	Healthy	33
P6	Female	60	25	0.85	42.6	Healthy	33
P7	Male	67	31.9	1.06	39.8	Obesity	37
