# Supplementary material for: A spatiotemporal proteomic map of human adipogenesis
Source: Nat Metab. 2024 Apr 2;6(5):861–79. doi: 10.1038/s42255-024-01025-8 (PMC11132986; doi:10.1038/s42255-024-01025-8)
Supplement: Supplementary file 23 — Unprocessed western blots Extended Data Fig. 10. [file 42255_2024_1025_MOESM23_ESM.pdf]

Early Knockdown of C19orf12

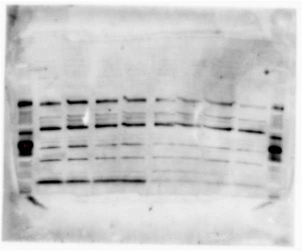

Proteintech, 27382-1-AP; Lot: 00053485

Marker overlay

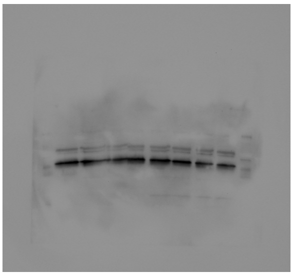

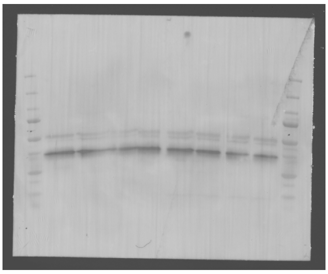

GAPDH (#2118, Cell Signaling) loading control

Late knock-down of C19orf12

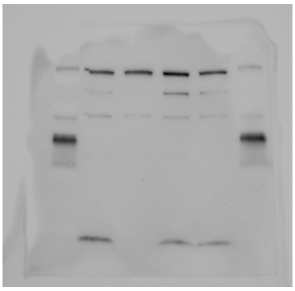

Proteintech, 27382-1-AP; Lot: 00053485

Marker overlay

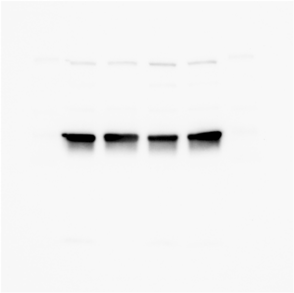

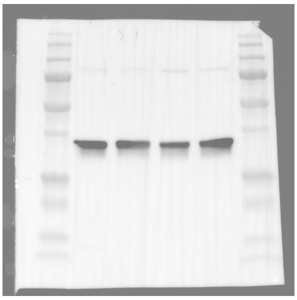

GAPDH (#2118, Cell Signaling) loading control

Full blots of Western Blot images in FIg. S10H.
